# Termination sequence between an inducible promoter and ubiquitous chromatin opening element (UCOE) reduces gene expression leakage and silencing

**DOI:** 10.1186/s13036-025-00499-8

**Published:** 2025-04-09

**Authors:** Tomoki Yanagi, Shean Fu Phen, Jonah Ayala, Deniz Ece Aydin, Susanna Jaramillo, David M. Truong

**Affiliations:** 1 Department of Biomedical Engineering, New York University (NYU) Tandon School of Engineering, Brooklyn, NY USA; 2 Department of Biology, New York University (NYU) Graduate School of Arts and Sciences, New York, NY USA; 3 Department of Pathology, NYU Grossman School of Medicine, New York, NY USA

**Keywords:** Synthetic biology, Genome engineering, Genome editing, Gene Silencing, Transdifferentiation

## Abstract

**Background:**

Inducible gene expression circuits enable precise control over target gene activation and are widely used in direct reprogramming. However, their usability is often compromised by DNA methylation-induced silencing, especially in iPSCs. This deactivates genetic circuits in engineered iPSCs preventing them from being used for long-term scalable expansion of desired cell types. A2-ubiquitous chromatin opening elements (A2UCOE) have been recognized for their anti-silencing properties, but they have not been used in human iPSCs with inducible systems for direct reprogramming. This study investigates the role of A2UCOE in inducible systems and identifies strategies to eliminate associated gene leakage enabling long-term use of engineered human iPSCs.

**Results:**

We developed a compact all-in-one gene circuit — containing a doxycycline-inducible Tet-On system, 863 bp of A2UCOE, and *FOXN1*, a transcription factor critical for thymic epithelial cell (TEC) differentiation — easily deployed to new genomic sites. However, we observed significant *FOXN1* gene leakage even without doxycycline, which is a novel limitation of A2UCOE. This leakage resulted in premature differentiation of iPSCs into TECs, limiting its continued use. To further investigate the relationship between A2UCOE and gene leakage, we generated A2UCOE fragments of varying lengths (1337 bp, 749 bp, and 547 bp) and found that all fragments, regardless of length, caused significant gene leakage. To solve this issue, we tested different spacer sequences between A2UCOE and the inducible promoter and found that the SV40 poly-A terminator fully eliminated *FOXN1* leakage, and we show this effect is not due to AT- or GC-content. Unexpectedly, this architecture further enhanced anti-silencing effects > 60% providing prolonged stability for at least 30 days.

**Conclusions:**

This study reveals a novel limitation of A2UCOE in inducible systems, specifically its contribution to gene leakage, which compromise sensitive systems like direct reprogramming of iPSCs. The inclusion of an SV40 poly-A sequence provides a practical solution and genomic architecture to improve the functionality of A2UCOE-based circuits. It also suggests investigating how termination of transcription modulates gene silencing as a novel design parameter. These findings have significant implications for the design of robust gene circuits, particularly in applications involving iPSCs, regenerative medicine, and cell therapy.

**Graphical abstract:**

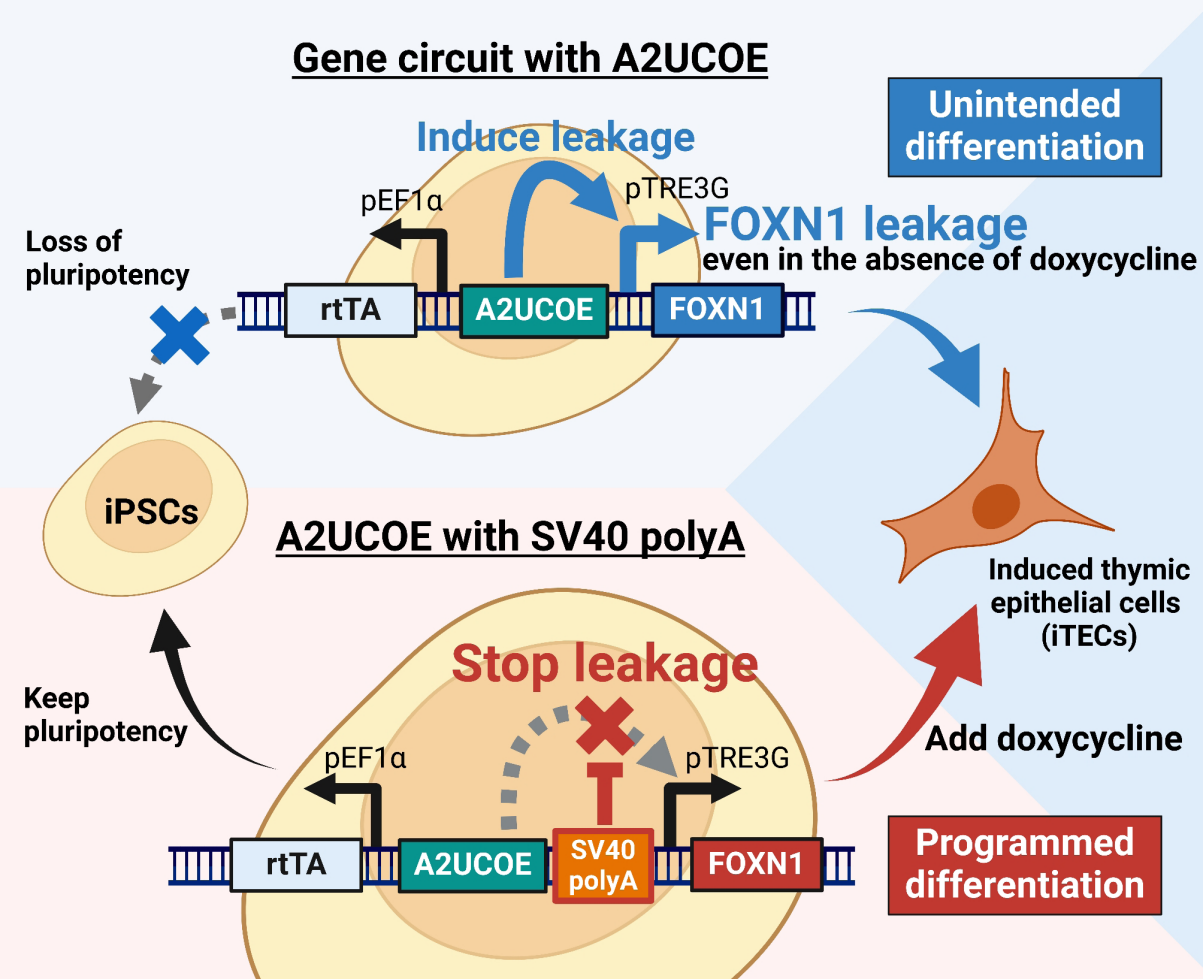

**Supplementary Information:**

The online version contains supplementary material available at 10.1186/s13036-025-00499-8.

## Background

Mammalian inducible gene expression systems enable control of target genes in response to cell-permeable molecules such as doxycycline and are widely utilized across many biological disciplines. They are particularly essential in the field of direct reprogramming. Direct reprogramming or transdifferentiation is an emerging technology enabling direct conversion of one cell type to another through forced overexpression of master transcription factors [1]. This technique has been increasingly applied to hasten the induction of renewable cells like induced pluripotent stem cells (iPSCs) into desired somatic cells, called forward programming, since conventional directed differentiation approaches are lengthy and complex [2]. Critically for this, inducibility ensures that the iPSCs continue to grow renewably, before committing to a cell identity with limited capacity for expansion.

The most widely used inducible system is the reverse tetracycline-controlled transactivator (rtTA) system, commonly known as the Tet-On system [3, 4]. In the Tet-On system, exogenously applied doxycycline complexes to rtTA enabling it to bind and activate gene expression at Tet binding sites found in synthetic tetracycline response element (TRE) promoters. However, the Tet-On system is entirely dependent on stable expression of rtTA within cells [5, 6], as changes in rtTA expression levels reduce or even eliminate output [7]. Transgene silencing of rtTA, presumably through DNA methylation, has been a common problem, especially in iPSCs, which express high levels of de novo DNA methyltransferases [8, 9].

Most direct reprogramming Tet-On gene circuits utilize a split design, where the rtTA components are expressed from a constitutive promoter integrated at one location, while the target genes under the rtTA-responsive promoters are integrated at another location [10]. Genomic integrations typically utilize lentivirus particles, which have a limited DNA size capacity (< 10 kb) and integrate randomly in the genome [11, 12]. Random integration often results in position-effect variegation of expression and complete silencing over time [13, 14]. Site-specific integration into safe-harbor genomic sites like ROSA26 or AAVS1 via homology-directed repair help mitigate this in a clonal manner [15], but the clones eventual succumb to transgene silencing effects. Alternatively, “all-in-one” versions of the Tet-On system, which join the constitutive and inducible elements in a bidirectional transcriptionally opposing configuration, enable a compact circuit with more stable expression [10].

Even so, these systems often include insulator elements to further mitigate silencing. The A2-ubiquitous chromatin opening element (A2UCOE) has been shown to reduce gene silencing when paired to diverse constitutive promoters and in different cell types [16–18]. The A2UCOE element is located on human chromosome 7, between the housekeeping genes heterogeneous nuclear ribonucleoprotein A2/B1 (*HNRNAPA2B1*) and chromobox 3 (*CBX3*), which are transcribed in opposite directions. The A2UCOE region spans approximately 3 kb and contains a large CpG island, which is unusual in its density of CpG dinucleotides. While most CpG islands attract DNA methylation, the CpG density in the A2UCOE is thought to resist methylation by maintaining an open chromatin structure, thereby helping prevent gene silencing [19–21]. Further studies have revealed shorter lengths of A2UCOE, from 2.2 kb to 0.6 kb, that also effectively reduce silencing [16, 17, 21–31]. While it remains unclear which length is most effective for anti-silencing, these shorter A2UCOE fragments have promise in compact gene circuits such as those used in gene therapy.

Here, we constructed an “all-in-one” Tet-On system utilizing the A2UCOE that is optimized for site-specific genome integration through homology-directed repair. As a model for studying transcription dynamics, we integrated and expressed the pioneer transcription factor *FOXN1* in human iPSCs. When constitutively overexpressed, *FOXN1* is a powerful pioneer factor sufficient to force fibroblasts and iPSCs into thymic epithelial cells (TECs), which make up the thymus where T-cells are matured [32–34]. We hypothesized that an inducible *FOXN1* expression system would enable a renewable source of human TECs for pre-clinical studies. However, our studies revealed that while the A2UCOE does reduce silencing of the constitutively expressed portions of the construct, it also causes leaky expression of the TRE promoter directed genes.

## Materials and methods

### ATAC-seq analysis using external database data

ATAC-seq data was obtained from series GSE170231, which hosts publicly available chromatin accessibility profiles [35]. Fastq data sets for ATAC-seq on human cell line PGP1 were downloaded and integrated into our analysis. Following data retrieval, quality control checks were conducted, including assessments of read depth and signal-to-noise ratio to ensure data integrity. Sequencing reads were aligned to the human reference genome GRCh38 using Bowtie 2. Peak calling was performed using IGV.

### Cell culture

Human iPSC line PGP1 was obtained from George Church’s laboratory. Cells were grown at 37 °C at 5% CO2 and ambient (~ 19%) oxygen in B8 iPSC medium [36], which contains DMEM/F12 (Corning, NY, USA), 200 µg/mL of L-Ascorbic acid 2-phosphate, 5 µg/mL of human insulin (Gibco), 5 µg/mL of human transferrin (InVitria), 20 ng/mL of sodium selenite, 40 ng/mL of fibroblast growth factor 2-G3 (FGF2-G3) (made at Northwestern University's core facility), 0.1 ng/mL of neuregulin 1 (NRG1) (Peprotech), and 0.1 ng/mL of transforming growth factor beta-3 (TGFb3) (Peprotech), on plates coated with Cultrex (R&D systems, Inc, MN, USA), and they were grown until achieving about 70% confluence. Passaging of cells was performed by clump passaging using 0.5 mM EDTA, and with Accutase (Innovative Cell Technologies) when single cell dissociation was required. Cells were assayed regularly for mycoplasma.

### Construction of genetic circuits

The bidirectional Tet-On plasmid system was assembled from DNA fragments obtained from different source plasmids available from Addgene or by chemical synthesis from Integrated DNA Technologies (IDT, USA). Cloning was performed using a mixture of standard restriction enzyme cloning and Gibson assembly. The plasmid backbone is based on the yeast vector pRS416 (Boeke laboratory) and contains E. coli replication elements including the ampicillin resistance marker. Various lengths of UCOE fragments, including a 0.9 kb-UCOE, were either synthesized by IDT or PCR amplified from human genomic DNA. The *FOXN1* gene was extracted from the plasmid provided by Addgene (Addgene#101445). These genes were assembled into genetic circuits using Gibson assembly with E. coli strains (TOP10, EPI300, and dam-/dcm- competent cells) as well as yeast-based assembly. The constructed gene circuits were subsequently verified for sequence accuracy through full-length sequencing performed by Plasmid-EZ service (Azenta, USA).

### Genetic circuit integration

For targeted genetic circuit integration into the Rogi1 site on chromosome 1, we designed guide RNAs (gRNAs) to specifically cleave this region. These gRNAs were expressed using a plasmid based on the PX459 vector (Addgene#62988). Next, homology arms (HAs) of approximately 450 bp each were designed for the 5’ and 3’ sides of the Rogi1 cleavage site and amplified by PCR to create DNA fragments. For one-pot Golden Gate cloning, we combined 10 ng of the 5’ HA, 10 ng of the 3’ HA, 200 ng of the *FOXN1*-containing all-in-one Tet-On plasmid, 1 µL of BsaI, 1 µL of T4 DNA ligase, 1 µL of T4 ligation buffer (10x), and 1 µL of ATP, adjusting the total volume to 10 µL with distilled water. The reaction mixture was incubated overnight at 37 °C. Then, to prepare for homology-directed repair (HDR), the plasmid containing the homology arms was linearized using restriction enzymes (Kpn1 and Nhe1) at the ends of the homology arms. The sequences of the gRNAs and homology arms are provided in Supplemental Table 1. The linearized DNA, along with the plasmid expressing the gRNAs, was introduced into cells via nucleofection using the P3 Primary Cell 4D-Nucleofector kit (Lonza). Following nucleofection, cells were subjected to selection with 3 µg/mL of blasticidin to ensure the survival of only those cells that successfully integrated the desired genetic constructs. After the selection process, the accuracy of the genetic editing was verified using junction PCR, a technique designed to confirm the presence of the gene-editing event at the intended genomic location. The primer sequences used for this PCR are listed in Supplemental Table 2.

### Cell differentiation protocol

Once cell confluency reached approximately 40–50%, the culture medium was switched to differentiation medium. The differentiation medium consisted of Advanced DMEM supplemented with 60 mg/L of 2-ascorbic acid 2-phosphate, 10 mL/L of GlutaMAX™, and 15 mL/L of 1 M HEPES (Gibco). To induce differentiation, 1 µg/mL of doxycycline was added to the medium. Cells were then cultured in this condition for a period of 7 days to promote differentiation.

### Counting RFP-positive cells by flow cytometry

Cells were detached using Accutase and fully dissociated into a single-cell suspension. The cell count was determined using the Countess 3 (Thermo Fisher), and the cells were resuspended at a concentration of 5,000 cells/µL in 1 mM EDTA-PBS solution. The suspension was then passed through a 35 μm filter to ensure complete dissociation into individual cells. Flow cytometry was performed using a BD Accuri C6 flow cytometer (Becton Dickinson Company, NJ, USA). A total of 10,000 cells were analyzed per sample, and the number of RFP-positive cells was measured using the PE fluorescence channel. The data were analyzed using Floreada.io.

### RNA extraction and cDNA synthesis

RNA was extracted from cells at 80–90% confluence using the Quick-RNA Miniprep Kit (Zymo Research, CA, USA), following the manufacturer’s protocol. The quantity of extracted RNA was then measured using a NanoDrop spectrophotometer (Thermo Fisher Scientific, MA, USA). Subsequently, reverse transcription via MMLV-RT was performed using ABScript Neo RT Master Mix (ABclonal, Inc., MA, USA) to synthesize cDNA according to the instructions provided by the manufacturer.

### Quantitative real-time PCR (qPCR) analysis

Quantitative PCR was performed using the Forget-Me-Not™ EvaGreen^®^ qPCR Master Mix manufactured by Biotium (CA, USA). All samples were prepared according to the manufacturer’s instructions. To ensure reproducibility and accuracy, each sample was measured in technical duplicates or triplicates, and the results were averaged. The expression levels of the target genes were normalized to the housekeeping gene *RPS29* using the Ct values. Relative gene expression was calculated using the ΔΔCT method. The list of primers used is provided in Supplemental Table 3.

### FOXN1 leakage analysis

We evaluated *FOXN1* gene leakage using qPCR. The *FOXN1* coding sequence used in this study was derived from a plasmid obtained from Addgene (Addgene #101445). To analyze gene leakage, we designed three primer sets specific to the synthetic *FOXN1* gene using Primer-BLAST (https://www.ncbi.nlm.nih.gov/tools/primer-blast/) (Supplemental Fig. 1). qPCR was performed on three experimental groups: parental PGP1 cells, bulk iTEC cells containing the integrated 0.9 UCOE-*FOXN1* gene circuit, and bulk cells with the integrated 0.9 UCOE-*FOXN1* gene circuit treated with doxycycline. Among the three primer sets, Primer Set 1, which produced the least variation in Ct values and the most consistent amplification curve, was selected and used throughout this study.

### Immunofluorescence staining

For immunofluorescence, cultured cells were first aspirated to remove the supernatant and then washed with phosphate-buffered saline (PBS). Cells were fixed by adding 4% paraformaldehyde (PFA) and incubated for 30 min at room temperature. After fixation, the cells underwent three washes with PBS. Blocking was performed using 1% bovine serum albumin (BSA) in PBS with 0.1% Tween 20 (PBST) for 30 min to prevent non-specific binding. DLL4 polyclonal antibody (#PA5-97664, Thermo Fisher Scientific, MA, USA) was diluted at 1:200 in 1% BSA in PBST and applied to the cells, followed by overnight incubation at 4 °C. After incubation, cells were washed three times with PBS. Goat anti-Rabbit IgG (H + L) Cross-Adsorbed Secondary Antibody, Alexa Fluor™ 488 (Thermo Fisher Scientific, MA, USA) was then diluted at 1:2000 in 1% BSA in PBST and applied to the cells. The cells were incubated for 1 h in the dark at room temperature, followed by washing with PBS. Stained cells were imaged using the EVOS™ M7000 Imaging System (Thermo Fisher Scientific, MA, USA).

### Design and creation of various A2UCOE fragments

Using the UCSC Genome Browser (https://genome.ucsc.edu/), we obtained information on the A2UCOE region, including neighboring gene sequences, promoters, and enhancers (Supplemental Fig. 2). Based on this information, we designed the 1.3 UCOE, 0.9 UCOE, 0.7 UCOE, and CBX UCOE fragments. The 0.9 UCOE fragment was amplified by PCR from cDNA generated based on the human genome using the following primers: forward primer (5′-TGAAATTAACGCCGACGGGAG-3′) and reverse primer (5′-GAGGGGAGCGGAGAACCG-3′). The 1.3 UCOE fragment was constructed by synthesizing specific segments through Integrated DNA Technologies (IDT, USA) and assembling the complete gene circuit using Gibson Assembly. The sequences of each A2UCOE fragment are provided in Supplemental Table 4.

### Design and creation of random AT-Rich spacer sequences

To create random sequences, we used ChatGPT-4 to generate 238 bp sequences with approximately 35%, 50%, and 65% AT content. These gene fragments were synthesized by Integrated DNA Technologies (IDT, USA). The sequence information for each spacer is provided in Supplemental Table 5.

### Statistical analysis

Statistical analyses were conducted using GraphPad Prism 10.2.2 software. For comparisons between two groups, unpaired t-tests were used to determine statistical significance. For analyses involving three or more groups, one-way ANOVA was performed. These methods were chosen based on the distribution of the data and the number of comparisons required. The level of significance was set at *p* < 0.05 for all tests.

### Creating figures and charts

Figures and charts were created using GraphPad Prism 10.2.2, Adobe Illustrator, Microsoft PowerPoint and BioRender. Each software tool was selected for its specific capabilities in graphical representation and design to ensure high-quality visual data presentation. Images were not altered except to enhance brightness, contrast, or introduce pseudo-color for fluorescence.

## Results

### Development of the “all-in-one” Tet-On system

**Fig. 1:**
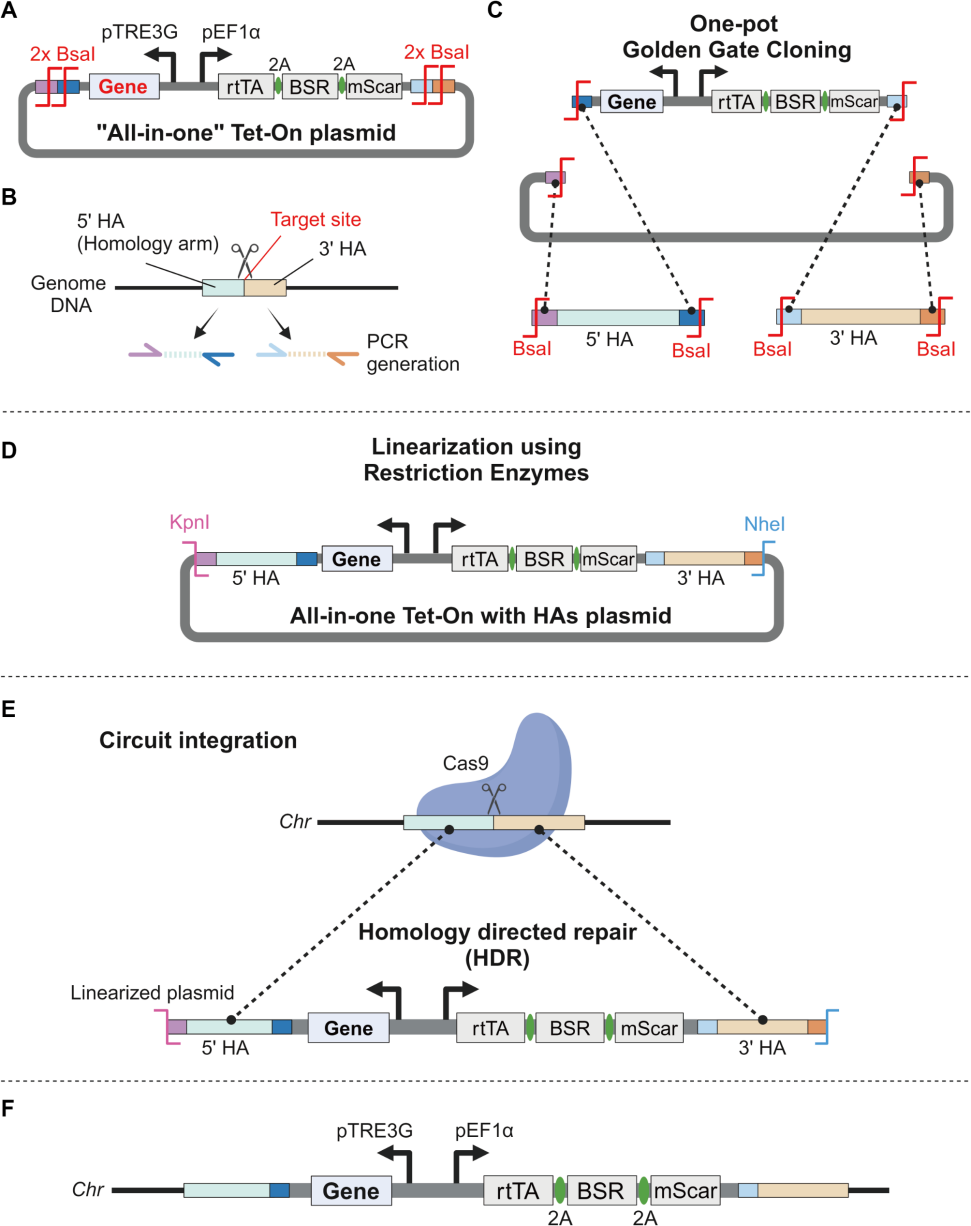
Design and integration of the “all-in-one” Tet-On system plasmid for targeted gene knock-in. A. Schematic representation of the all-in-one Tet-On plasmid. The human EF1α promoter drives the expression of the reverse tetracycline transactivator (rtTA), along with the blasticidin resistance (BSR) gene and mScarlet (mScar) reporter gene. The TRE3G promoter is oriented in the opposite direction to control the expression of the target gene (Gene). Two sets of adjacent BsaI restriction sites (2x BsaI) flank the regulatory elements. B. Generation of homology arms (HAs) through PCR amplification. The 5’ and 3’ homology arms, each approximately 450 bp, are designed to match the target genomic site for precise knock-in through homologous recombination. C. One-pot Golden Gate Cloning is used to insert the homology arms into the all-in-one plasmid. The BsaI restriction enzyme is used to ligate the homology arms with the Tet-On gene circuit. D. The plasmid (All-in-one Tet-On with HAs plasmid) is linearized using KpnI and NheI restriction enzymes to prepare it for integration into the genome. The final construct includes the homology arms and the regulatory elements of Tet-On system. E. Schematic of the targeted circuit integration. The linearized plasmid and a Cas9-gRNA vector are co-electroporated into mammalian cells. Cas9 creates a double-stranded break at the target site, and the homology arms direct homologous recombination (HDR), enabling precise knock-in of the genetic circuit into the desired genomic location. F. Final structure of the genomic integration, with the All-in-one Tet-On gene circuit at the target site

We developed an “all-in-one” Tet-On system, a gene circuit containing all Tet-On control mechanisms on a single plasmid, suitable for straightforward knock-in at target genomic regions (Fig. 1A). Downstream of the human EF1α promoter, we placed the rtTA gene, which controls gene transcription by binding to the TRE3G promoter, along with a blasticidin resistance (BSR) gene for selection and the mScarlet (mScar) reporter gene. The TRE3G promoter is positioned in the opposite orientation to the EF1α promoter, enabling the insertion of a target gene downstream of the TRE3G promoter for regulated expression of a gene of interest. The TRE3G promoter allows for target gene expression in the presence of both rtTA and doxycycline.

To facilitate precise genomic integration, we incorporated two pairs of adjacent BsaI restriction sites flanking the regulatory elements on the plasmid to allow for scarless Golden Gate cloning (Fig. 1A). One first identifies the intended cleavage site in the target region using CRISPR/Cas9 and then designs homology arms (HA) ~ 450 bp for the 5’ and 3’ regions, which can be amplified by PCR (Fig. 1B). These amplified HAs are then inserted to flank the all-in-one Tet-On plasmid via one-pot Golden Gate cloning (Fig. 1C). The plasmid, linearized using KpnI and NheI, is then co-electroporated with a Cas9-gRNA-expressing plasmid into mammalian cells to induce homology-directed repair (HDR) into the desired genomic region (Fig. 1D-F). This all-in-one plasmid allows for controlling the expression of target genes in potentially any genomic region using doxycycline.

### 0.9 UCOE enhances long-term gene expression in gene-edited iPS cells

**Fig. 2:**
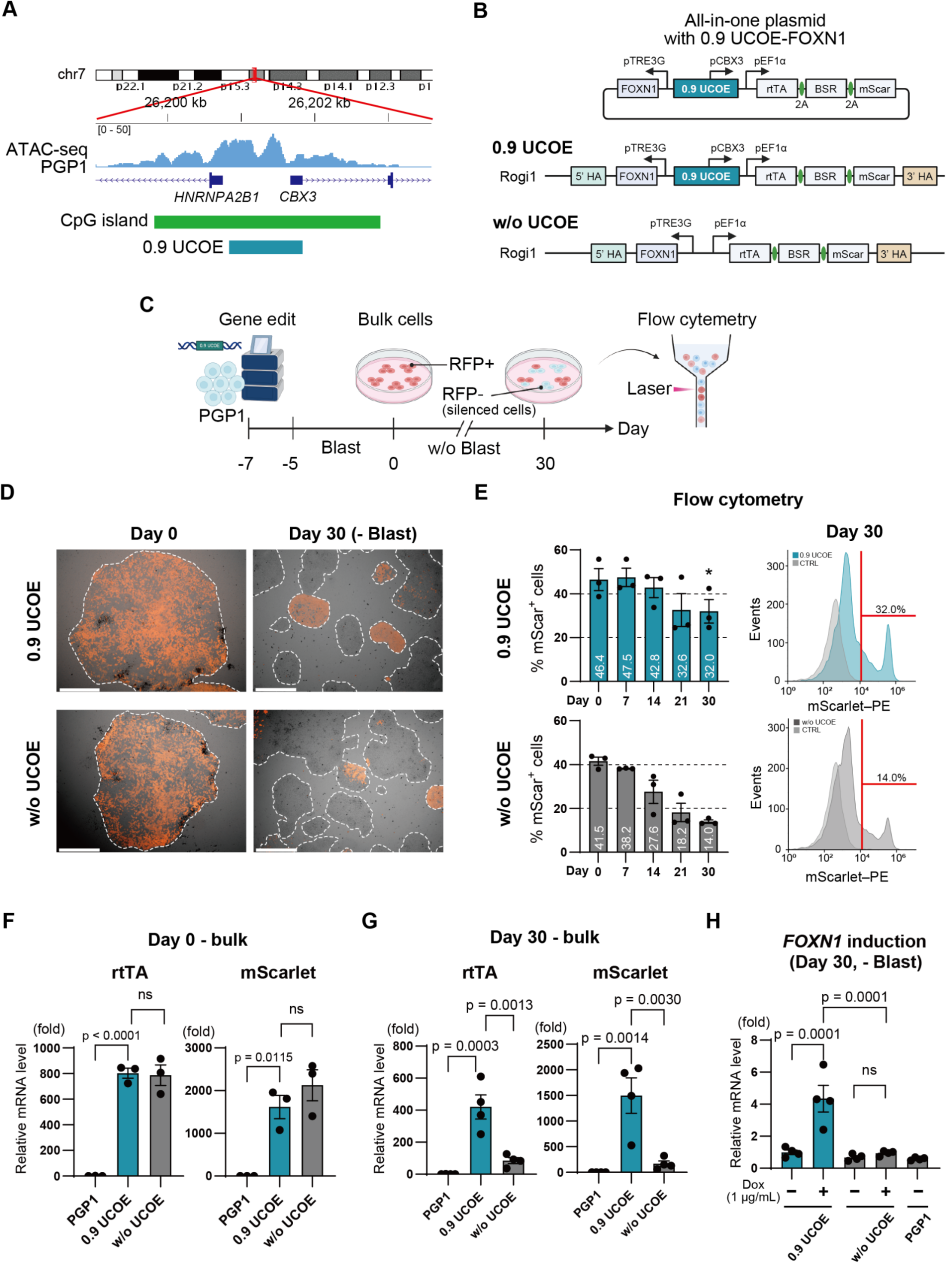
The 0.9 UCOE enhances long-term gene expression stability in gene-edited iPS cells. **A**. Location and design of the 0.9 UCOE fragment within the A2UCOE region on human chromosome 7. ATAC-seq data from PGP1 iPSCs show transcriptionally active regions overlapping with a CpG island between the *HNRNAPA2B1* and *CBX3* genes, suggesting open chromatin in this area. The 0.9 UCOE, isolated from this region, spans 863 bp, including exon 1 of *CBX3* and part of the *HNRNAPA2B1* promoter. **B**. Schematic of the all-in-one Tet-On plasmid design with and without the 0.9 UCOE. The 0.9 UCOE was placed upstream of the EF1α promoter to drive stable expression of rtTA and selection markers. **C**. Experimental workflow for generating and analyzing bulk iPSC populations after gene editing. PGP1 iPSCs were edited using CRISPR/Cas9, followed by 5 days of blasticidin selection. Flow cytometry analysis was performed periodically over the 30-day culture period to monitor the percentage of RFP-positive (mScarlet+) cells. **D**. Fluorescence microscopy images of the 0.9 UCOE and w/o UCOE bulk populations on Days 0 and 30. **E**. Quantification of RFP-positive cells by flow cytometry in the 0.9 UCOE and w/o UCOE bulk populations over time (*n* = 3). **F**. Reverse Transcriptase Quantitative PCR (RT-qPCR) analysis of transcription levels for rtTA and mScarlet in bulk populations on Day 0 (*n* = 3). **G**. RT-qPCR analysis of rtTA and mScarlet transcription levels in bulk populations on Day 30 after 30 days of culture without blasticidin (*n* = 4). **H**. RT-qPCR analysis of induced *FOXN1* transcription in response to doxycycline on Day 30 after 30 days of culture without blasticidin (*n* = 4). P-values were calculated using a two-tailed unpaired t-test and one-way analysis of variance with Tukey’s honestly significant difference test. Data are presented as mean ± SEM. For detailed data, statistical analyses, and exact p-values, see source data file

To minimize gene silencing and promote stable long-term expression of rtTA and the selectable markers, we placed a 0.9 kb A2UCOE upstream of the constitutive human EF1α promoter (Fig. 2A, Supplemental Table 4). The A2UCOE is located on human chromosome 7, between the *HNRNAPA2B1* and *CBX3* genes, and has a large CpG island [19, 20]. We analyzed ATAC-seq data of this region in PGP1 iPSCs from the ENCODE consortium [35], and observed peaks corresponding to the CpG island, suggesting high transcriptional activity in the same region within PGP1 cells (Fig. 2A). To enable the generation of thymic epithelial cells (TECs), we then introduced a codon-optimized human *FOXN1* cDNA sequence into the construct (Fig. 2B) [32, 37]. TECs are critical players in both positive and negative selection of precursor T-cells during central tolerance and adaptive immunity [38]. This genetic circuit was integrated into a safe harbor region on chromosome 1 (Rogi1) of PGP1 human iPSCs using CRISPR/Cas9-assisted HDR [39, 40], along with a control genetic circuit lacking UCOE (w/o UCOE). After a 2-day rest period following genetic editing, cell integrants were selected for with 3 µg/mL blasticidin for 5 days. Successful integration of the genetic circuits was confirmed by PCR amplification of the newly generated 5’ and 3’ integration junctions at the Rogi1 target region (Supplemental Fig. 3). Total iPSC colonies were pooled to generate a bulk population.

We then evaluated the stability of gene expression in the bulk-gene edited population of cells (Fig. 2C), which we note will contain both targeted and untargeted integrants. Starting from the day blasticidin selection was completed (Day 0), the bulk cells were cultured for 30 days in medium without blasticidin to permit silencing, and the stability of gene expression downstream of UCOE was evaluated by quantifying the number of RFP-positive (mScarlet+) cells via flow cytometry (Fig. 2C). According to fluorescence microscopy, most colonies and cells were RFP-positive on Day 0, but by Day 30 visibly showed a reduction in RFP due to silencing (Fig. 2D). Flow cytometry analysis revealed no significant difference in the percentage of RFP-positive cells (46.4% vs. 41.5%) in the starting Day 0 cells (Fig. 2E). The low fraction of RFP-positive cells in the initial population is likely due to clump passaging of human iPSCs, which may protect some cells from antibiotic selection. Nevertheless, by Day 30, the percentage of RFP-positive cells was significantly higher in the 0.9 UCOE population (32.0%) compared to the w/o UCOE population (14.0%) (Fig. 2E).

Additionally, we evaluated the transcription levels of the introduced genes, rtTA and mScarlet. On Day 0, both rtTA and mScarlet gene transcription levels were approximately 800-fold and 2000-fold higher, respectively, in both the 0.9 UCOE bulk and w/o UCOE bulk groups compared to unmodified parental PGP1 cells. However, there was no significant difference in transcription levels between the 0.9 UCOE and w/o UCOE bulk groups on Day 0 (Fig. 2F). In contrast, on Day 30, while the expression levels of rtTA and mScarlet decreased compared to Day 0 in the 0.9 UCOE group, they remained approximately 400-fold and 1500-fold higher, respectively. In the w/o UCOE group, a substantial decline in expression levels was observed, making them far less usable (Fig. 2G).

More importantly, the bulk UCOE cells after 30 days still responded to doxycycline administration showing significantly enhanced expression of the target gene, *FOXN1*, whereas by comparison the w/o UCOE group on Day 30 had no response and expression (Fig. 2H). These results suggest that the A2UCOE promotes long-term stable gene expression in gene-edited iPS cells and helps ensure that cells continue to respond to doxycycline for inducing target genes in the Tet-On system in the absence of selection.

### Establishment of pre-induced thymic epithelial cell line and differentiation into induced thymic epithelial cells (iTEC)

**Fig. 3:**
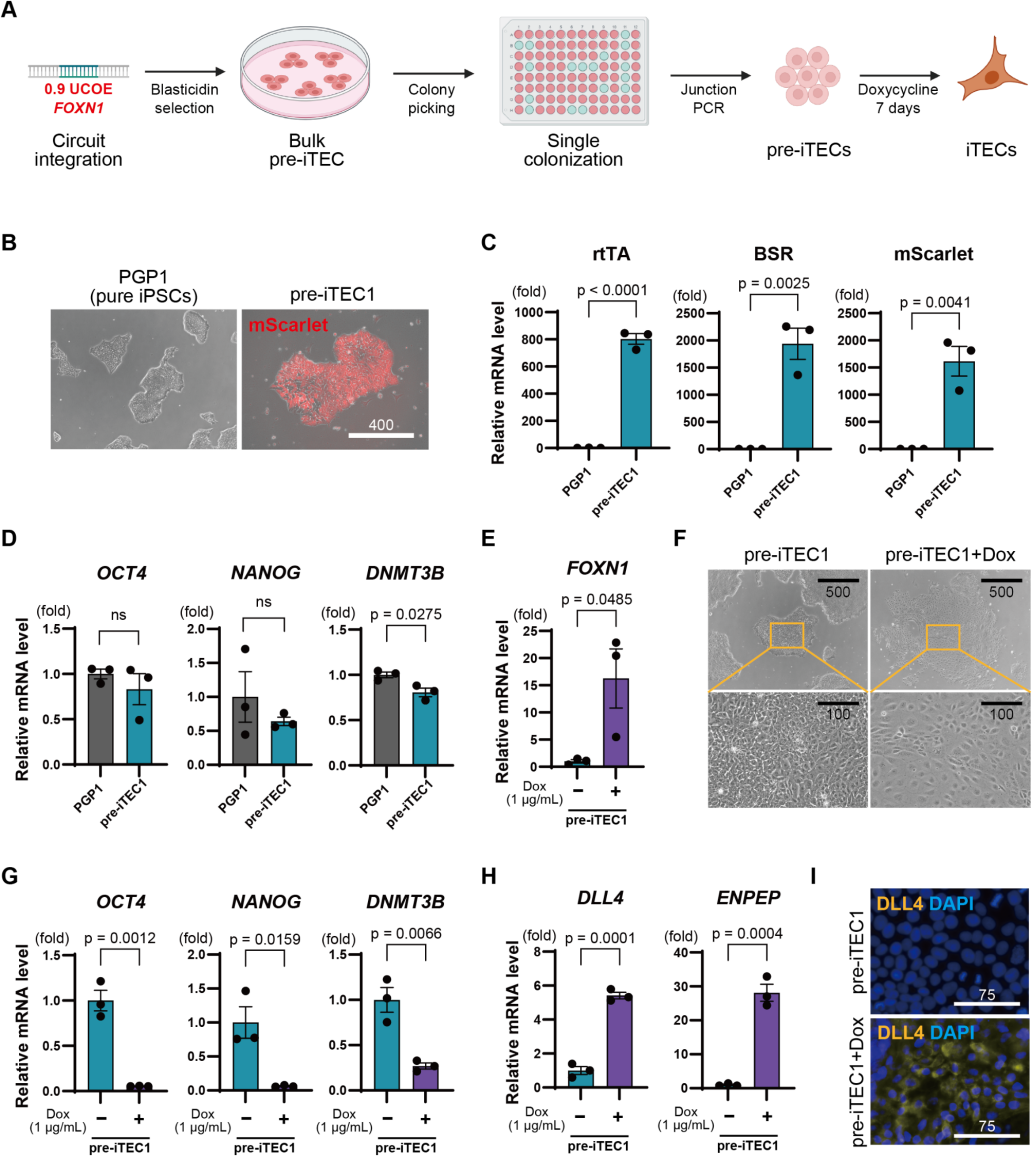
Establishment of pre-induced thymic epithelial cell (pre-iTEC) line and induction of differentiation into thymic epithelial cells (iTEC). **A**. Experimental scheme: Genetic circuits were introduced into iPSCs to create pre-induced thymic epithelial cells (pre-iTEC). Induction into induced thymic epithelial cells (iTEC) was achieved by administering 1 µg/mL doxycycline for 7 days. **B**. Representative images of the created pre-iTEC1 and PGP1 cells. The RFP signal indicates the expression of the mScarlet gene. **C**. RT-qPCR comparison of the introduced genes rtTA, BSR, and mScarlet between groups (*n* = 3). **D**. RT-qPCR comparison of undifferentiated marker genes *OCT4*, *NANOG*, and *DNMT3B* in PGP1 cells and pre-iTEC1 (*n* = 3). **E**. RT-qPCR comparison of *FOXN1* gene expression in pre-iTEC1 and pre-iTEC1 treated with doxycycline groups (*n* = 3). **F**. Morphological changes in pre-iTEC1 upon doxycycline administration. **G**. RT-qPCR comparison of undifferentiated markers in pre-iTEC1 with and without doxycycline treatment (*n* = 3). **H**. Comparison of TEC markers *DLL4* and *ENPEP* (Ly51) in pre-iTEC1 upon doxycycline treatment (*n* = 3). **I**. Representative image of immunofluorescence staining for DLL4 in pre-iTEC1 treated with doxycycline. P-values were calculated using a two-tailed unpaired t-test. The data are presented as mean ± SEM. For detailed data, statistical analyses, and exact p-values, see source data file

Next, we sought to isolate single-cell-derived clones with the *FOXN1* gene circuit containing the UCOE, for stably making induced thymic epithelial cells (iTEC), using the above protocol (Fig. 3A). A total of 24 colonies were picked, and PCR confirmed the correct insertion of the genetic circuit in 9 of these cell lines indicating a 37% integration efficiency (Supplemental Fig. 4). From these, we selected one cell line and established it as our pre-induced thymic epithelial cell line 1 (pre-iTEC1). This cell line exhibited a dense and flat colony morphology indicative of the primed pluripotent state similar to the parental PGP1 cells and strongly expressed mScarlet fluorescent protein (Fig. 3B). Reverse Transcriptase quantitative PCR (RT-qPCR) revealed significant expression of rtTA, BSR, and mScarlet compared to PGP1 cells (Fig. 3C). Additionally, the expression of the pluripotency markers *OCT4*, *NANOG*, and *DNMT3B* were comparable to that in PGP1 cells, indicating that the undifferentiated state was maintained (Fig. 3D).

Next, we evaluated the forward programming potential of pre-iTEC1 iPSCs into iTECs. Upon treatment with doxycycline (1 µg/mL, 24 h), *FOXN1* expression was significantly induced in the doxycycline-treated group, as confirmed by RT-qPCR (Fig. 3E). Moreover, when 1 µg/mL of doxycycline was administered for 7 days in B8-iPSC media, which enforces the pluripotent state, the morphology of the cells changed from the dense colony-like appearance characteristic of iPSCs to a looser and elongated form (Fig. 3F, Supplemental Fig. 5), indicating that pre-iTEC cells underwent morphological changes upon doxycycline treatment even in the presence of media meant to maintain pluripotency. The expression of the pluripotency markers *OCT4*, *NANOG*, and *DNMT3B* was significantly decreased in the doxycycline-treated group (Fig. 3G). Furthermore, we observed an increase in expression of the key TEC markers *DLL4* and *ENPEP* (Ly51) in the doxycycline-treated group (Fig. 3H). Immunostaining further revealed strong DLL4 protein expression in the doxycycline-treated group (Fig. 3I). These results demonstrate that pre-iTEC1 iPSCs reprogram into iTECs upon *FOXN1* induction alone.

### 0.9 UCOE induces gene leakage and premature differentiation of iPS cells

**Fig. 4:**
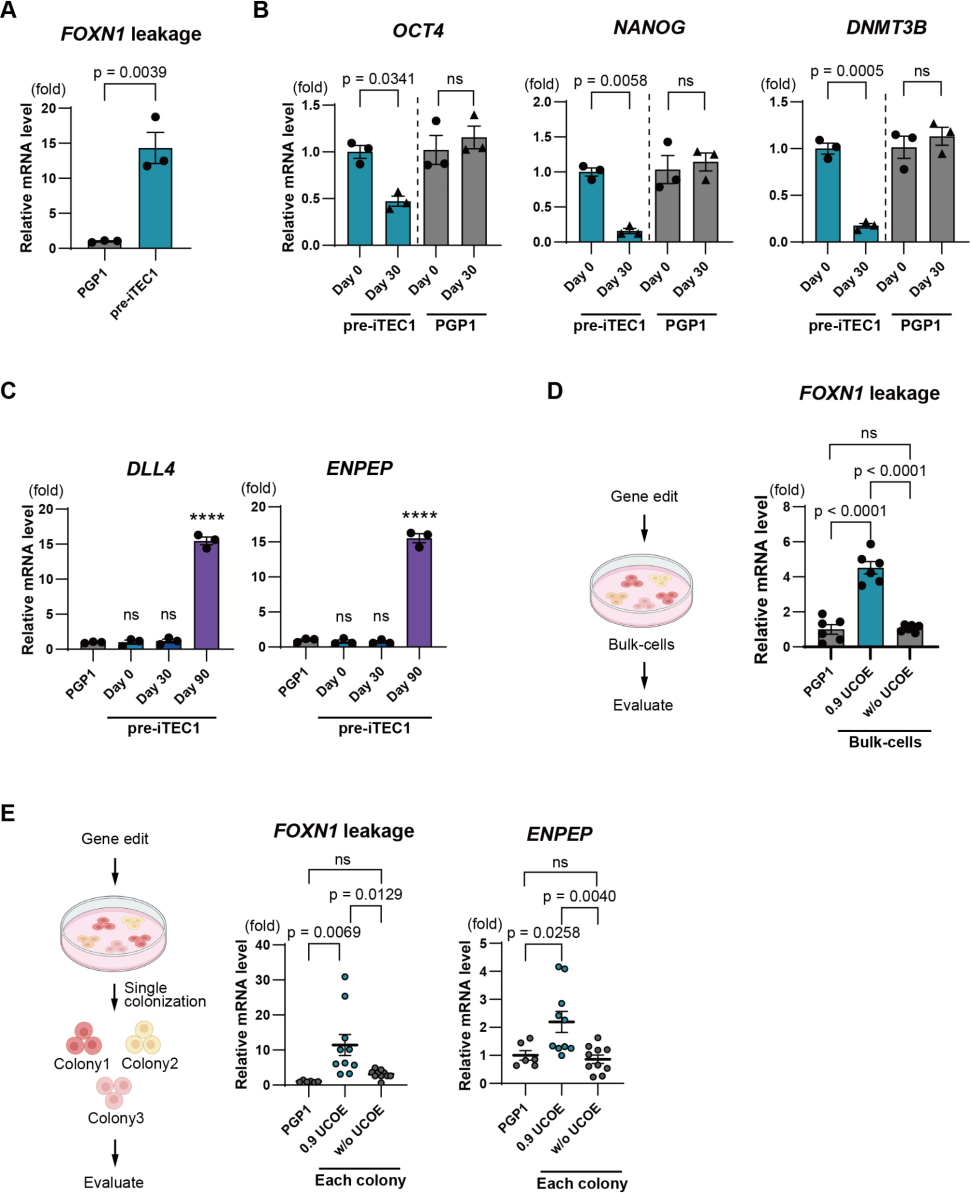
0.9 UCOE induces gene leakage and unintended differentiation of iPS cells. **A**. Analysis of *FOXN1* gene leakage in pre-iTEC1 and PGP1 without doxycycline treatment (*n* = 3). **B**. RT-qPCR comparison of undifferentiated marker genes *OCT4*, *NANOG*, and *DNMT3* in pre-iTEC1 and PGP1 cultured without doxycycline for 30 days (*n* = 3). **C**. RT-qPCR comparison of TEC-specific markers *DLL4* and *ENPEP* (Ly51) in pre-iTEC1 during 90 days culture without doxycycline (*n* = 3). **D**. Analysis of *FOXN1* leakage in bulk cell populations of the 0.9 UCOE and w/o UCOE group by RT-qPCR (*n* = 3). **E**. *FOXN1* leakage and *ENPEP* (Ly51) expression analysis in single-cell-derived colonies of 0.9 UCOE group and w/o UCOE group by RT-qPCR (*n* = 10). P-values were calculated using a two-tailed unpaired t-test and one-way analysis of variance with Tukey’s honestly significant difference test. Data are presented as mean ± SEM. For detailed data, statistical analyses, and exact p-values, see the source data file

However, we observed *FOXN1* gene leakage in the pre-iTEC1 cell line even in the absence of doxycycline (Fig. 4A). When pre-iTEC1 was cultured without doxycycline for 30 days, we noted a significant decrease in the pluripotency markers *OCT4*, *NANOG*, and *DNMT3B*, indicating premature differentiation, whereas PGP1 cells maintained their pluripotency over the same 30-day culture period (Fig. 4B). Furthermore, after long-term culture for 90 days without doxycycline, we observed increased transcription of the TEC-specific markers *DLL4* and *ENPEP* (Ly51), despite the absence of doxycycline (Fig. 4C). We also observed morphological changes after 30 days as the w/o UCOE bulk group maintained a dense colony-like appearance like iPSCs, whereas some cells in the 0.9 UCOE bulk group exhibited a looser and elongated form like TECs (Fig. 3F, Supplemental Fig. 6).

To test the hypothesis that A2UCOE caused gene leakage, we integrated both 0.9 UCOE and w/o UCOE genetic circuits into PGP1 cells, generated new bulk groups, and evaluated the level of *FOXN1* leakage in each group. The w/o UCOE group showed no significant difference in *FOXN1* leakage compared to the control PGP1 cells, while the UCOE group exhibited significantly higher *FOXN1* leakage in the absence of doxycycline (Fig. 4D). Next, we compared single-cell-derived clones from both groups. Ten colonies each were picked from both the 0.9 UCOE and w/o UCOE groups, and precise integration at Rogi1 was confirmed by PCR (Supplemental Fig. 7). When comparing *FOXN1* leakage among these clones, no significant *FOXN1* leakage was observed in the w/o UCOE colonies. In contrast, the 0.9 UCOE colonies displayed significant *FOXN1* leakage and greater heterogeneity in absolute expression from clone to clone (Fig. 4E). Moreover, the 0.9 UCOE colonies showed elevated expression of the TEC marker *ENPEP*, even shortly after cell line establishment (Fig. 4E). These findings indicate that 0.9 UCOE contributes to gene leakage in the Tet-On system, leading to unexpected differentiation of iPS cells.

### Evaluation of gene stability and gene leakage using various lengths of A2UCOE fragments

**Fig. 5:**
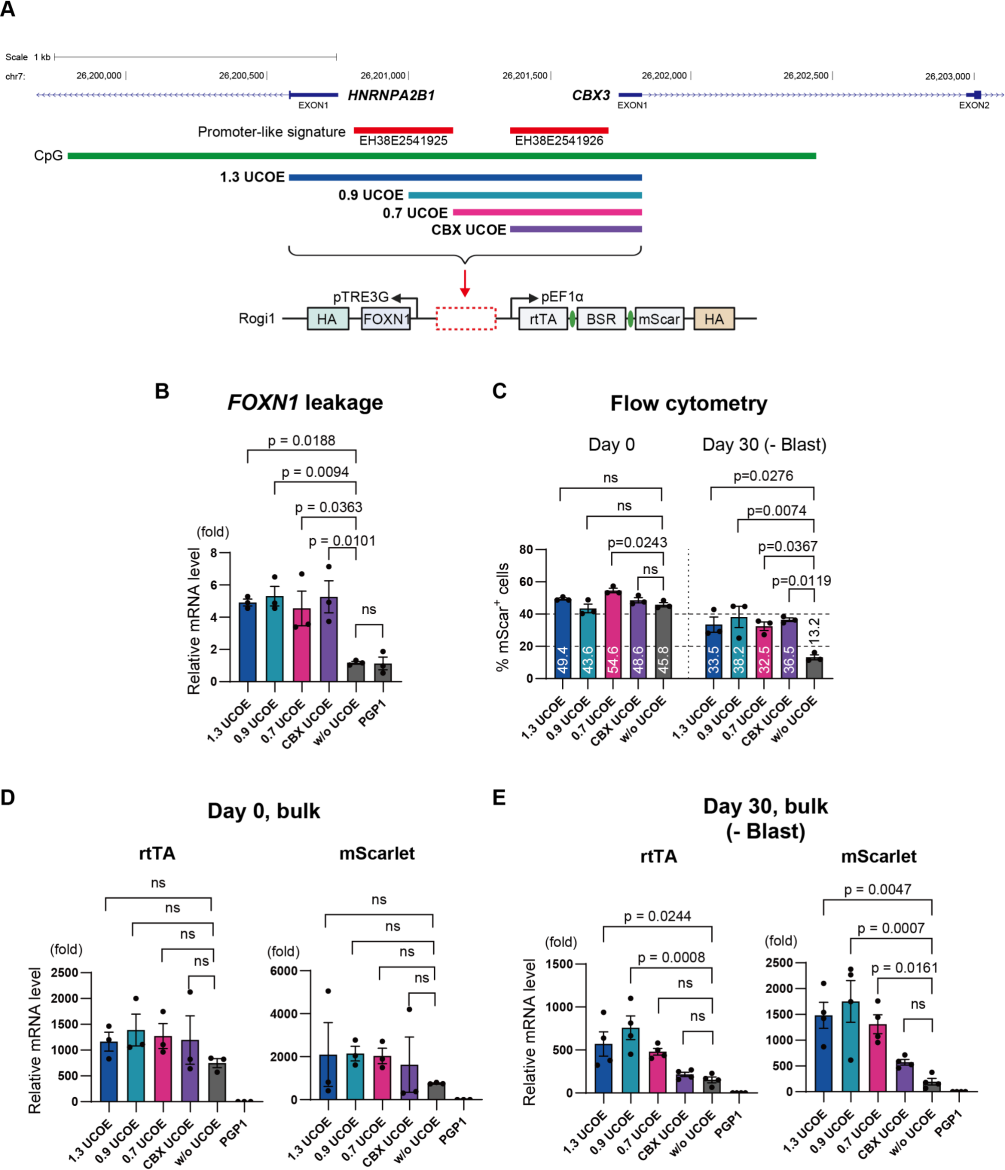
Evaluation of gene stability and gene leakage using various lengths of A2UCOE fragments. **A**. Design of different A2UCOE fragments. The 1.3 UCOE (1,337 bp) includes the untranslated region (UTR) and exon 1 of *HNRNAPA2B1*, as well as the promoter-like regions EH38E2541925 and EH38E2541926 extending to include the *CBX3* exon 1. The 0.9 UCOE (863 bp) included the part of the *HNRNAPA2B1* promoter and extending to exon 1 of *CBX3*. The 0.7 UCOE (749 bp) contains only the *CBX3* exon 1, promoter region, and intergenic sequence. The CBX UCOE (547 bp) includes only the *CBX3* promoter region and exon 1. These fragments were incorporated into the *FOXN1* Tet-On inducible gene circuit and integrated into the Rogi1 site of PGP1 iPSCs. B. *FOXN1* gene leakage in each UCOE group assessed on Day 0 by RT-qPCR (*n* = 3). **C**. Flow cytometry analysis of RFP-positive cells in each UCOE group on Day 0 and Day 30 (*n* = 3). **D**. Transcription levels of rtTA and mScarlet on Day 0 in each UCOE group by RT-qPCR (*n* = 3). **E**. Transcription levels of rtTA and mScarlet on Day 30 in each UCOE group after 30 days of culture without blasticidin by RT-qPCR (*n* = 3). P-values were calculated using one-way analysis of variance with Tukey’s honestly significant difference test. The data are presented as mean ± SEM. For detailed data, statistical analyses, and exact p-values, see source data file

Next, we generated several A2UCOE fragments to investigate whether longer or shorter forms could reduce gene leakage while enhancing expression stability. Previous studies have examined various lengths of A2UCOE fragments and their abilities to promote long-term stable expression; however, no studies to date have evaluated A2UCOE in terms of gene leakage. Using the UCSC Genome Browser, we identified the precise location of A2UCOE in the human genome along with information on nearby promoters and enhancers (Supplemental Fig. 2). Based on this information, we designed three new UCOE fragments (Fig. 5A, Supplemental Table 4). The 1.3 UCOE fragment, contains the untranslated region (UTR) and exon 1 of *HNRNAPA2B1*, along with EH38E2541925, a promoter-like element associated with *HNRNAPA2B1*, extending to include EH38E2541926, a promoter region and exon 1 of *CBX3*. The 0.7 UCOE fragment, 749 bp in length, does not include the *HNRNAPA2B1* exon 1 or promoter region but contains the *CBX3* exon 1 and promoter region, as well as the intergenic region between EH38E2541925 and EH38E2541926. The CBX UCOE fragment is shorter, at 547 bp, and includes only the *CBX3* promoter region and exon 1. These three different UCOE fragments were then used to generate new *FOXN1* expressing human iPSC clones as before.

We then compared *FOXN1* gene leakage and constitutive marker gene expression silencing across the different UCOEs. First, we found that on Day 0 all UCOE fragments exhibited significant *FOXN1* gene leakage at comparable levels (Fig. 5B). Using flow cytometry, we then found that on Day 0, the percentages of RFP-positive cells were similar across all UCOE fragments (49.4% for 1.3 UCOE, 43.6% for 0.9 UCOE, 54.6% for 0.7 UCOE, and 48.6% for CBX UCOE). Among these, only the 0.7 UCOE group showed a significant difference compared to the w/o UCOE control group, while the other UCOE fragments did not show any significant differences. By Day 30, the percentages of RFP + cells were also comparable across UCOEs (32.5–38.2%) (Fig. 5C, Supplemental Fig. 8).

We also evaluated the transcription levels of rtTA and mScarlet. On Day 0, transcription levels of rtTA and mScarlet were comparable across all UCOE fragments, with no significant differences relative to the w/o UCOE control group (Fig. 5D). However, by Day 30, transcription levels of rtTA were significantly maintained in the 1.3 UCOE and 0.9 UCOE groups compared to the w/o UCOE group. For mScarlet, transcription levels were significantly maintained in the 1.3 UCOE, 0.9 UCOE, and 0.7 UCOE groups compared to the w/o UCOE group. Notably in the 0.5 CBX UCOE group, transcription levels of both rtTA and mScarlet had decreased to levels comparable to those in the w/o UCOE group (Fig. 5E).

These results indicate that an A2UCOE length of at least 0.7 kb positively influences the maintenance of transcription levels, whereas the CBX-only UCOE fragment is insufficient to sustain transcription. However, when assessing the RFP-positive cell number via flow cytometry, no significant differences were observed between UCOE fragments of 0.7 kb or longer and the shorter CBX UCOE. Nevertheless, examining fluorescence output revealed that the intensity peak for the CBX-UCOE shifted significantly to the left compared to the peaks of the other three UCOE fragments, suggesting that the CBX UCOE results in a higher number of weakly positive RFP cells (Supplemental Fig. 9). Additionally, when doxycycline was administered to the CBX UCOE group after 30 days of culture, *FOXN1* transcription levels were significantly higher compared to the w/o UCOE group and comparable to the 0.9 UCOE group (Supplemental Fig. 10). This finding suggests that, despite the lower transcription and protein expression levels of rtTA in the CBX UCOE group, the inducible gene expression function was stably maintained over an extended period. Overall, these results demonstrate that any UCOE fragment can sustain inducible gene expression function over time compared to the condition without UCOE.

### SV40 poly-A spacer sequences mitigate gene leakage caused by A2UCOE

**Fig. 6:**
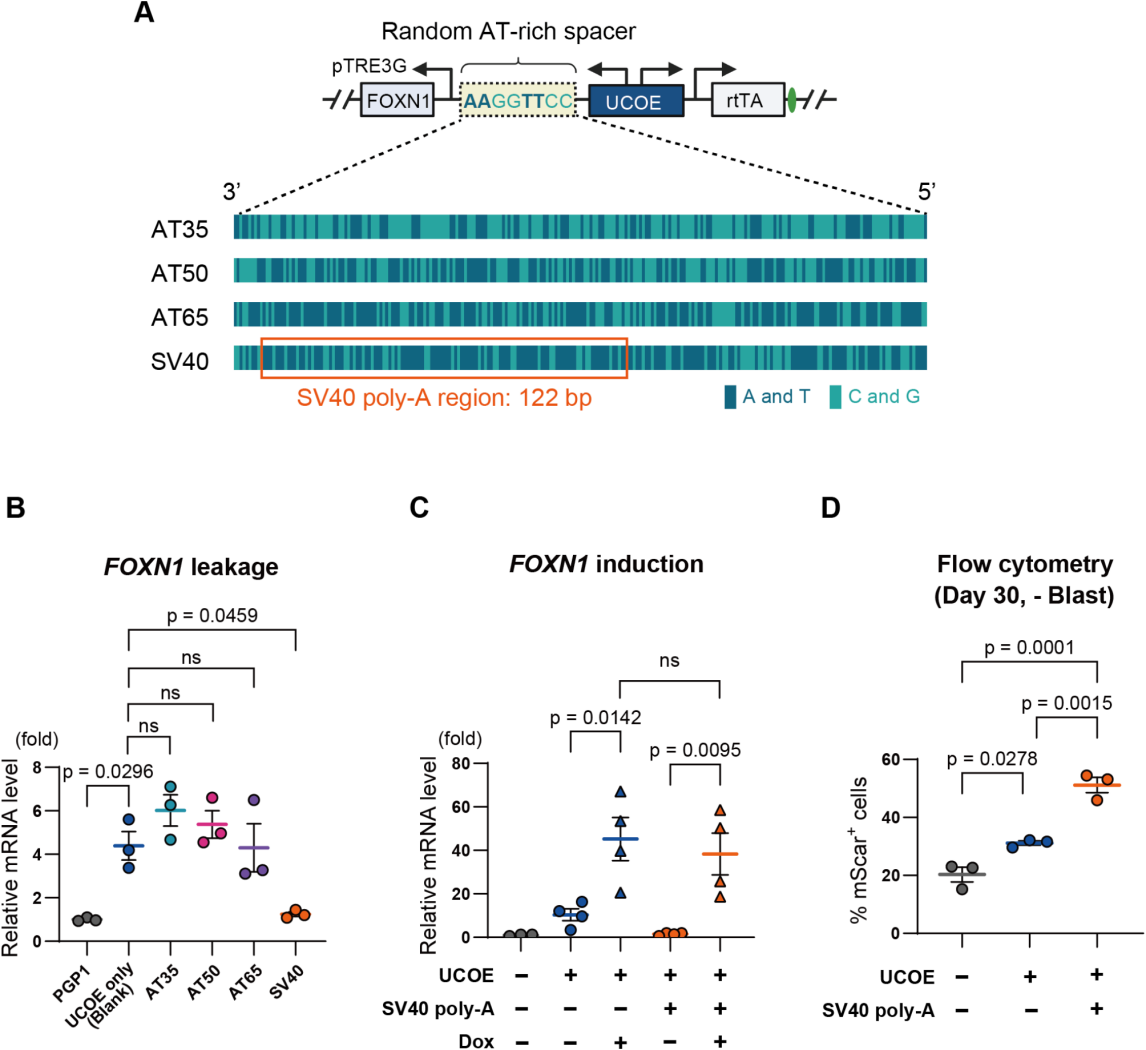
SV40 poly-A spacer sequences mitigate gene leakage caused by A2UCOE. **A**. Schematic of genetic circuit architecture with various AT-rich spacer sequences between A2UCOE and the TRE-*FOXN1* promoter. Spacer sequences were designed randomly with 238 bp lengths and varied AT content: about 35% (AT35), 50% (AT50), and 65% (AT65), as well as a 65% AT-rich spacer containing the 122 bp SV40 poly-A termination sequence (SV40). The dark green and light green bars represent the sequence composition, with A and T shown in dark green and C and G in light green. The SV40 poly-A sequence is highlighted with an orange box. **B**. Quantification of *FOXN1* gene leakage in bulk cell populations following the integration of gene circuits with different AT-rich spacers by RT-qPCR (*n* = 3). **C**. Quantification of *FOXN1* gene induction in the bulk cell populations after 5 days of culture with blasticidin, followed by doxycycline treatment (*n* = 4). **D**. Flow cytometry analysis of RFP-positive (mScarlet+) cells in the SV40 poly-A spacer group after 30 days of culture without blasticidin (*n* = 3). P-values were calculated using one-way analysis of variance with Tukey’s honestly significant difference test. The data are presented as mean ± SEM. For detailed data, statistical analyses, and exact p-values, see source data file

Finally, we set out to solve the gene leakage caused by A2UCOE. Since UCOE sequences facilitate open chromatin in bidirectional gene clusters, we considered the possibility that it stabilizes RNA polymerase II elongation in both directions, despite the inducible TRE promoter lacking bound rtTA activating proteins. Whereas the UCOE contains a high density of CpG dinucleotides thought to prevent silencing, AT-rich sequences are thought to condense chromatin and resist protein binding, and are often found in termination sequences, matrix attachment regions, and replication origins. We hypothesized that inserting an AT-rich spacer sequence between the A2UCOE and TRE promoter might reduce the leaky gene expression. To test this hypothesis, we introduced several AT-rich spacer sequences between A2UCOE and TRE-*FOXN1*. We designed random 238 bp spacer sequences with evenly distributed AT content of 35%, 50%, and 65%, labeled AT35, AT50, and AT65, respectively (Fig. 6A, Supplemental Table 5). Additionally, we included a 238 bp spacer sequence with 65% AT content that contained the 122 bp termination sequence SV40 poly-A (SV40), which includes homopolymeric A and T tracts known to terminate transcription by forming hairpin structures and recruiting termination proteins.

These genetic circuits were integrated into the Rogi1 site in PGP1 cells as before, followed by a 5-day blasticidin selection to generate bulk populations. We then evaluated *FOXN1* gene leakage in these populations. As shown in Fig. 6B, the SV40 spacer significantly reduced *FOXN1* leakage to nearly the same level as the control group. In contrast, the AT35, AT50, and AT65 spacers did not show a significant reduction in leakage compared to the UCOE alone, suggesting that a 238 bp separation alone is insufficient to mitigate leakiness, nor does AT content alone seem to influence gene leakage.

Then, we evaluated whether the SV40 poly-A sequence, which effectively eliminated leakage, would impact the drug-inducible gene expression function of the Tet-On system. After a 5-day blasticidin selection following gene editing, doxycycline (1 µg/mL) was administered to the bulk cell populations, and *FOXN1* induction was assessed. The results showed that the SV40 poly-A spacer group exhibited significant induction of *FOXN1* transcription in response to doxycycline (Fig. 6C). Moreover, there was no significant difference in the level of induced gene transcription between the SV40 poly-A spacer group and the UCOE-only group.

Next, we tested whether this new genetic circuit architecture containing the SV40 poly-A spacer preserved the original UCOE benefits of anti-silencing and enhanced transcriptional stability. As before, cells were cultured without blasticidin for 30 days, and RFP-positive (mScarlet+) cells were quantified by flow cytometry. Even in the group containing the SV40 poly-A spacer, the number of RFP-expressing cells was maintained on Day 30 compared to the w/o UCOE control group (Fig. 6D). Moreover, the SV40 poly-A group also showed significantly better maintenance of RFP + cell numbers compared to the UCOE-only group, enhancing anti-silencing by > 60% compared to UCOE-alone (Fig. 6D). These results demonstrate that the SV40 poly-A termination sequence effectively eliminates gene leakage caused by A2UCOE while also boosting its original anti-silencing and transcription-stabilizing benefits.

## Discussion

**Fig. 7:**
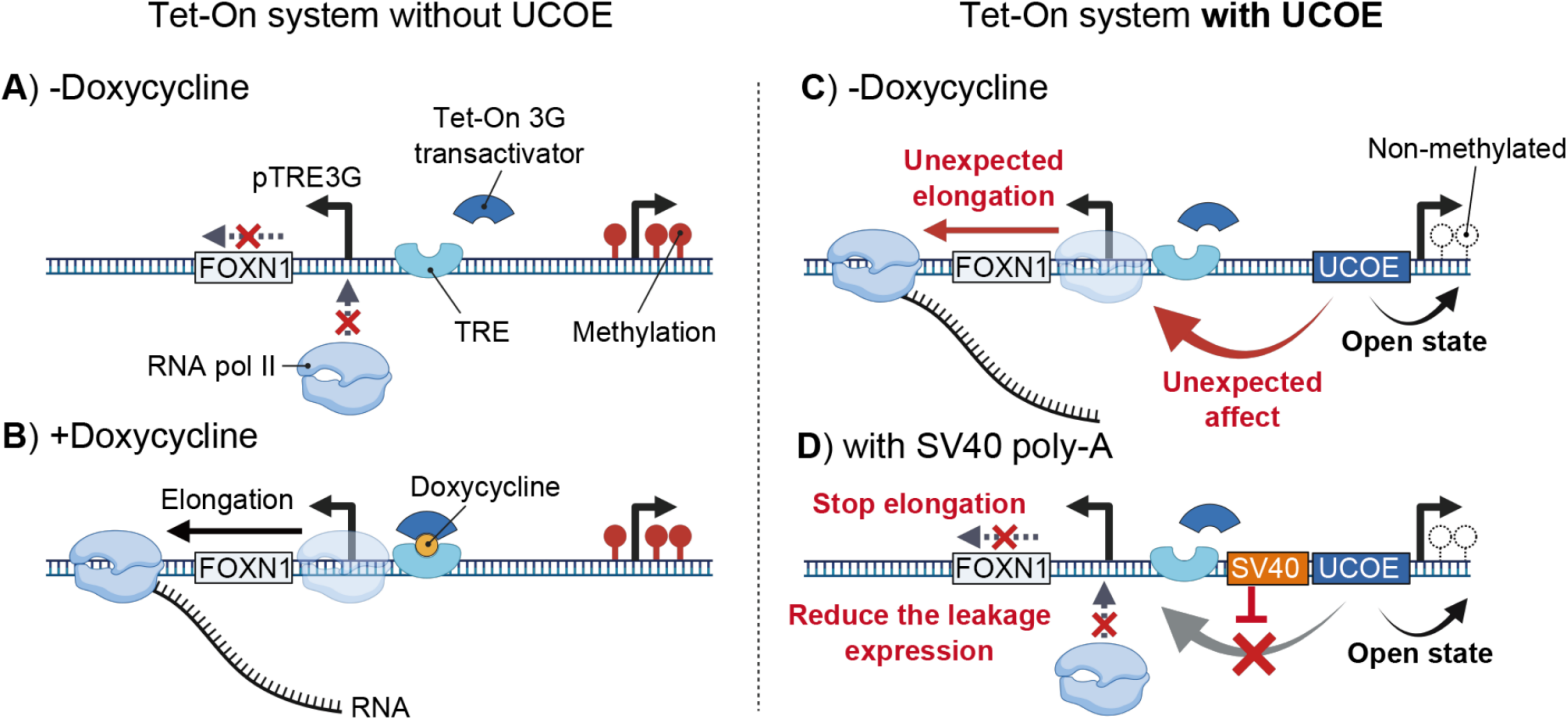
Mechanism of gene leakage in the Tet-On 3G system with A2UCOE. **A**. In the absence of doxycycline and A2UCOE, the Tet-On 3G transactivator does not bind to the tetracycline response element (TRE), and RNA polymerase II (RNA pol II) elongation does not initiate. The integrated gene will be silenced through methylation. **B**. In the presence of doxycycline, the Tet-On 3G transactivator binds to the TRE, initiating RNA pol II elongation and resulting in transcription. **C**. In an environment with A2UCOE but without doxycycline, the A2UCOE maintains the sense-strand constitutively expressed genes (rtTA, BSR, mScarlet) in an open chromatin state, preventing methylation and silencing. However, A2UCOE also exerts an unexpected effect on RNA pol II on the anti-sense strand, leading to unexpected elongation through the TRE promoter causing gene transcription even in the absence of doxycycline. **D**. When the SV40 poly-A sequence is present, it blocks the influence of A2UCOE on RNA pol II, stopping elongation and thereby reducing gene leakage

Here, we demonstrated that the A2UCOE causes leaky gene expression when paired with an all-in-one Tet-On system driving the master transcription factor *FOXN1*, leading to premature differentiation of iPSCs. The leaky expression is best explained by a model in which the open chromatin promoted by A2UCOE enables binding and elongation of RNA polymerase II (RNAPII) in both transcriptional directions without the aid of activating factors (Fig. 7). While we cannot rule out intrinsic promoter activity, transcription factor binding sites have not yet been found in A2UCOE [41]. We show that the leaky gene expression can be eliminated by inserting a spacer containing poly-A termination sequence, but not necessarily with AT-rich tracts alone.

While in vitro studies show that AT-rich tracts cause RNAPII pausing and termination, sometimes independent of specific protein factors [42–44], the specific poly-A termination signal was necessary to eliminate gene leakage. This likely means that recruitment of termination factors like poly-A polymerase and poly-A binding protein (PABPC) at canonical poly-A signal sequences are necessary for 3’-end processing and termination of basal transcription [45]. In fact, the SV40 spacer appeared to greatly enhance the anti-silencing effect of the UCOE (Fig. 6D). One possible explanation might be the blocking of spurious transcripts in the opposing orientation, as such transcripts can induce silencing by antagonizing replication [46]. We recommend then that when assembling synthetic genetic circuits with both inducible and constitutive promoters with the A2UCOE, that an SV40 poly-A sequence should be introduced between transcriptional units to block spurious RNAPII elongation. Further work might focus on the minimal number of spacer sequence elements that would confer these effects similar to the SV40 terminator.

We also introduce a user-friendly all-in-one Tet-On system that adopts a compact (5 kb) bidirectional design, where the EF1α promoter and TRE3G promoter are oriented in opposite directions. The A2UCOE with the SV40 poly-A terminator has minimal basal transcription, and resists silencing better than without. Compact Tet-On systems are particularly useful for lentiviral vector genomic integrations, which have a payload size limitation of 8–10 kb [11, 12]. However, our system utilizes site-specific integration via CRISPR/Cas9 assisted homology-directed repair into a recently characterized safe-harbor site Rogi1 [39]. While this safe-harbor site was reported to be better than other sites, it is still subject to epigenetic silencing, at least in human iPSCs. Indeed, we observed similar gene silencing frequencies over 15 to 29 days in experiments where we integrated the system lacking the SV40 poly-A at two other genomic sites, CYBB and TRBC1, indicating that susceptibility to epigenetic silencing is not limited to the Rogi1 safe-harbor site (Supplemental Fig. 11). Depending on the cell clone, we observed between 40 and 85.8% silencing, indicating clone-specific gene silencing. These results demonstrate that our all-in-one system is deployable across multiple genomic sites and suggest that epigenetic silencing is not inherently site dependent.

Another way to interpret why gene silencing occurs so frequently with constitutively expressed transgenes involves the cellular burden imposed by limited transcriptional and translational resources [47–49]. The EF1α promoter, for instance, naturally drives high expression of a translation elongation factor, essential for cellular function. When repurposed to constitutively express transgenes unnecessary from the cell’s perspective, this creates a fitness burden. Such fitness burdens may impose selective pressure on the host cell, potentially leading to transgene silencing as an adaptive response via epigenetic mechanisms. Although we did not directly investigate these effects on our integrated circuits through epigenomic methods, the general mechanisms underlying transgene silencing — such as heterochromatin spreading and CpG methylation — are well established [46]. Notably in human iPSCs, de novo DNA methyltransferases such as DNMT3B are highly active and could rapidly silence genes perceived to be burdensome (Fig. 3B). Thus, even with our anti-silencing circuit architecture, fitness-driven selection pressures toward transgene silencing are likely to persist and warrant further investigation.

The system is particularly useful when sustained inducible expression is required, but without any leaky basal expression [46]. This was particularly important when we expressed the pioneer transcription factor *FOXN1*, which was powerful enough to induce a thymic epithelial cell-like identity from leaky expression alone and in pluripotency media. Other direct reprogramming pioneer factors have the same ability to independently promote a new cell identity. Transcription factors such as *MYOD1* (muscle) [50], *NGN2* (neurons) [51], and *ETV2* (endothelial) [52], to name a few, also may cause premature differentiation in iPSCs if expression is not tightly controlled, at least when containing an A2UCOE. As mammalian synthetic biology progresses towards more practical applications where complex genetic circuits may be required, it will be important to further understand the rules by which transcription occurs based on the arrangement and architecture of DNA regulatory elements. The importance of spacing and orientation has been recently highlighted in modeling work of circuits similarly-sized to our system showing that transcriptional orientation can induce negative effects due to DNA supercoiling [53, 54].

A few limitations of this work are worth mentioning. First, we only evaluated A2UCOE’s anti-silencing properties with the constitutive human EF1α short promoter. While we did not experimentally test other common constitutive promoters (CAG, CMV, or PGK), previous reports indicate A2UCOE significantly improves gene expression stability across various promoter contexts (summarized in Supplemental Table 6) [16, 17, 21–30, 55, 56]. Future studies should examine promoter-UCOE combinations further, to refine and optimize circuit designs. A second limitation of our study is that the impact of A2UCOE on basal expression was only evaluated in the Tet-On system, and we did not test other inducible circuits. Recently, several new mammalian small molecule-inducible systems, such as the Cumate and synthetic zinc finger transcription regulators (synZiFTR) systems have been developed for controlled gene expression [57, 58]. It is possible that UCOE-induced gene leakage may behave differently in these systems due to differences in the promoter architecture. However, the Tet-On system is widely used and has undergone numerous improvements, making it one of the least leaky systems available. Despite using an improved Tet-On 3G system known for minimal leakage because it lacks transcription factor binding sites, we still observed UCOE-induced gene leakage. This suggests that UCOE could potentially have a negative impact on gene leakage in other drug-inducible circuits as well.

In summary, this study revealed that the A2UCOE can cause unintended gene leakage in bidirectional inducible gene circuits, leading to premature differentiation of iPS cells. This finding highlights a potential pitfall in the design of gene circuits for direct reprogramming, particularly when maintaining pluripotency is critical, such as in iPS and ES cells. This is also of extreme importance for therapeutic payloads where sustained expression and tight regulation are necessary for safety. Future designs of genetic circuits should consider the requirements needed for DNA found in between transcription elements, as is already naturally found in mammalian genomes.

## Electronic supplementary material

Below is the link to the electronic supplementary material.

Supplementary Material 1

Supplementary Material 2

Supplementary Material 3

## Data Availability

No datasets were generated or analysed during the current study.
