# Supplementary material for: Termination sequence between an inducible promoter and ubiquitous chromatin opening element (UCOE) reduces gene expression leakage and silencing
Source: J Biol Eng. 2025 Apr 9;19:29. doi: 10.1186/s13036-025-00499-8 (PMC11983960; doi:10.1186/s13036-025-00499-8)
Supplement: Supplementary file 3 — Supplementary Material 3 [file 13036_2025_499_MOESM3_ESM.docx]

**Supporting Information 1**

**Title: Termination sequence between an inducible promoter and ubiquitous chromatin opening element (UCOE) reduces gene expression leakage and silencing**

Tomoki Yanagi^1^, Shean Fu Phen^1,2^, Jonah Ayala^1^, Deniz Ece Aydin^1^, Susanna Jaramillo^1^, David M. Truong^1,3,*^.

^1^Department of Biomedical Engineering, New York University (NYU) Tandon School of Engineering, Brooklyn, NY, USA

^2^Department of Biology, New York University (NYU) Graduate School of Arts and Sciences, New York, NY, USA

^3^Department of Pathology, NYU Grossman School of Medicine, New York, NY, USA

^*^ Corresponding author: truond01@nyu.edu


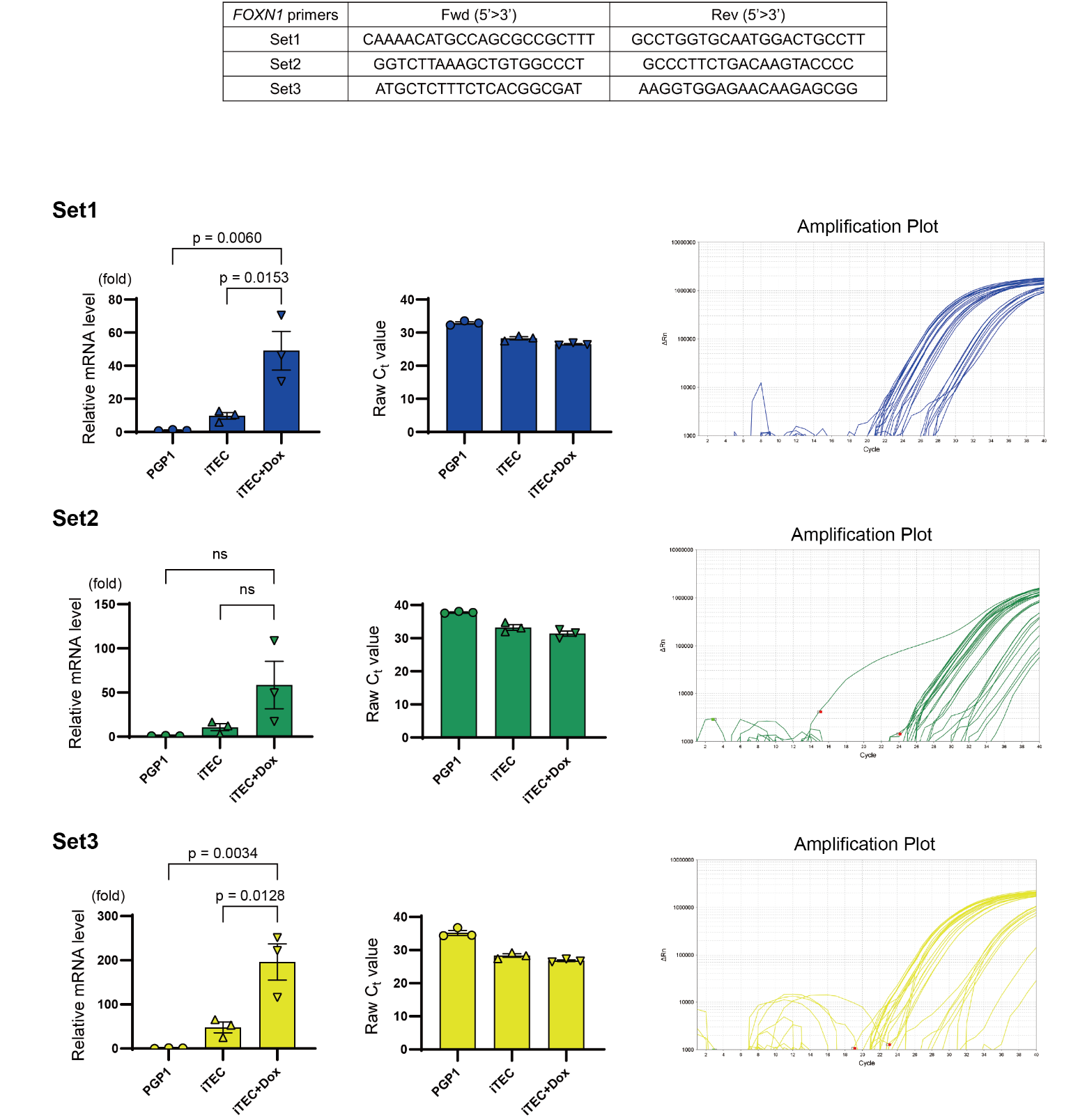


**Supplemental Figure 1. Evaluation of primer sets used for *FOXN1* leakage analysis.**

Three primer sets were designed for RT-qPCR targeting *FOXN1*. Relative mRNA levels normalized to the housekeeping gene *RPS29*, raw Ct values, and amplification plots are shown for each primer set.


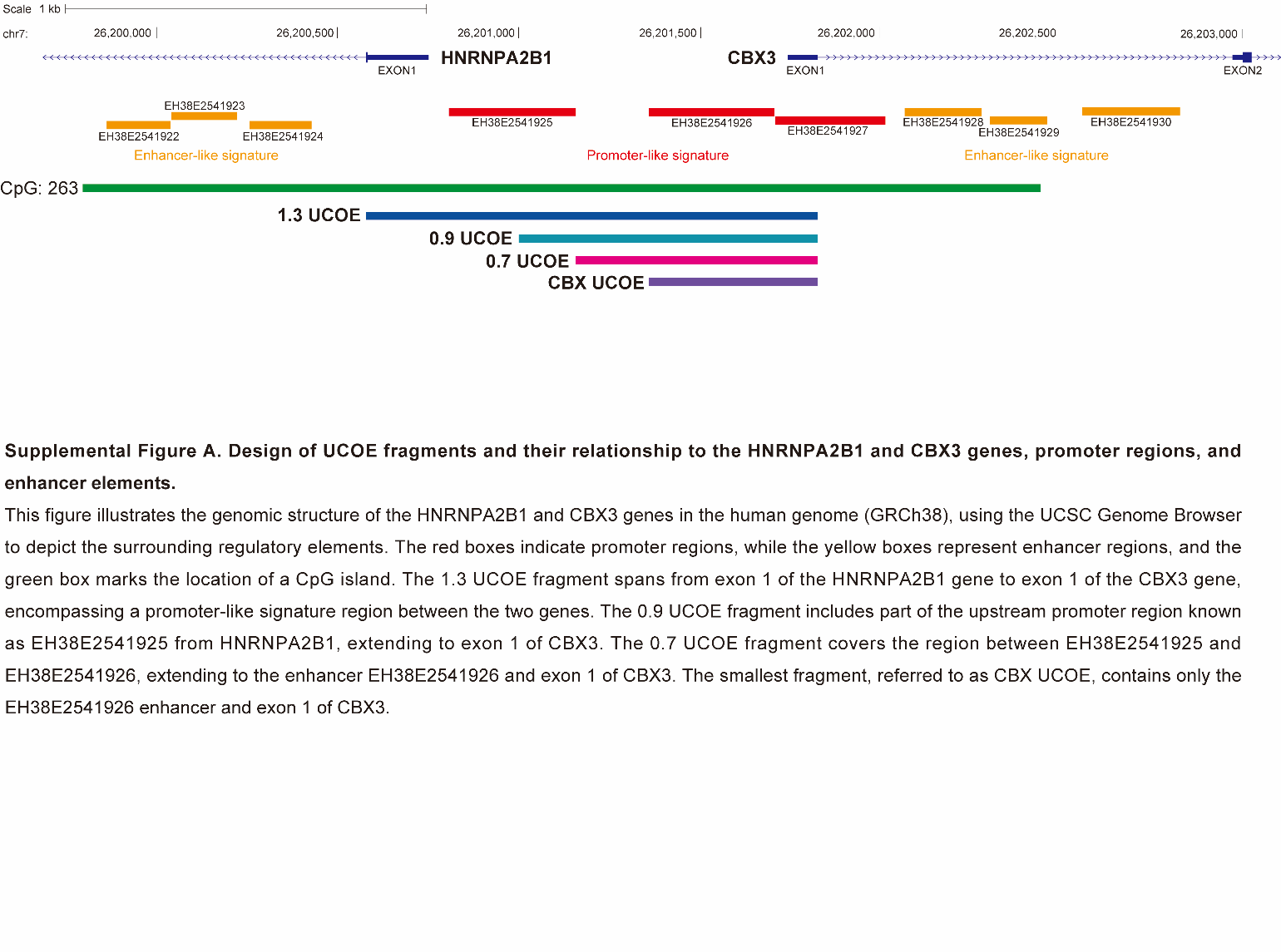


**Supplemental Figure 2. Design of UCOE fragments and their relationship to the *HNRNPA2B1* and *CBX3* genes, promoter regions, and enhancer elements.**

This figure illustrates the genomic structure of the *HNRNPA2B1* and *CBX3* genes in the human genome (GRCh38), using the UCSC Genome Browser to depict the surrounding regulatory elements. The red boxes indicate promoter regions, while the yellow boxes represent enhancer regions, and the green box marks the location of a CpG island. The 1.3 UCOE fragment spans from exon 1 of the HNRNPA2B1 gene to exon 1 of the *CBX3* gene, encompassing a promoter-like signature region between the two genes. The 0.9 UCOE fragment includes part of the upstream promoter region known as EH38E2541925 from *HNRNPA2B1*, extending to exon 1 of *CBX3*. The 0.7 UCOE fragment covers the region between EH38E2541925 and EH38E2541926, extending to the enhancer EH38E2541926 and exon 1 of *CBX3*. The smallest fragment, referred to as CBX UCOE, contains only the EH38E2541926 enhancer and exon 1 of *CBX3*.


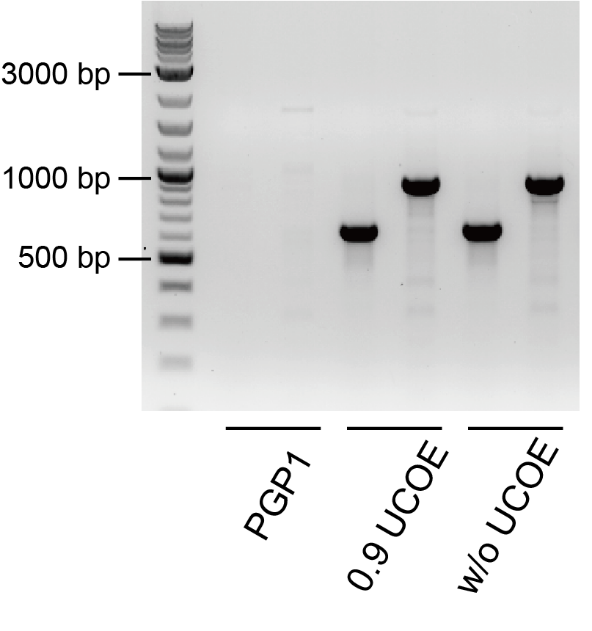


**Supplemental Figure 3. Junction PCR analysis of bulk cells with integrated gene circuits**

Integration of the 0.9 UCOE-FOXN1 gene circuit (0.9 UCOE) and the without UCOE-FOXN1 gene circuit (w/o UCOE) into the Rogi1 target region in bulk cell populations was confirmed using PCR. PCR amplification revealed 650 bp and 921 bp bands corresponding to the 5' and 3' integration junctions, respectively.


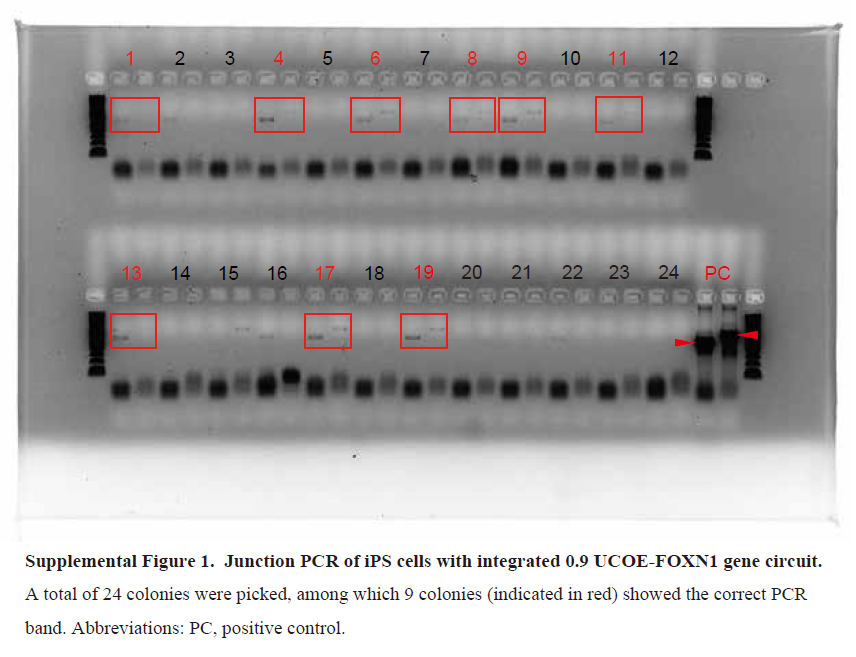


**Supplemental Figure 4. Junction PCR of iPS cells with integrated 0.9 UCOE-*FOXN1* gene circuit.**

A total of 24 colonies were picked, among which 9 colonies (indicated in red) showed the correct PCR band. Abbreviations: PC, positive control.


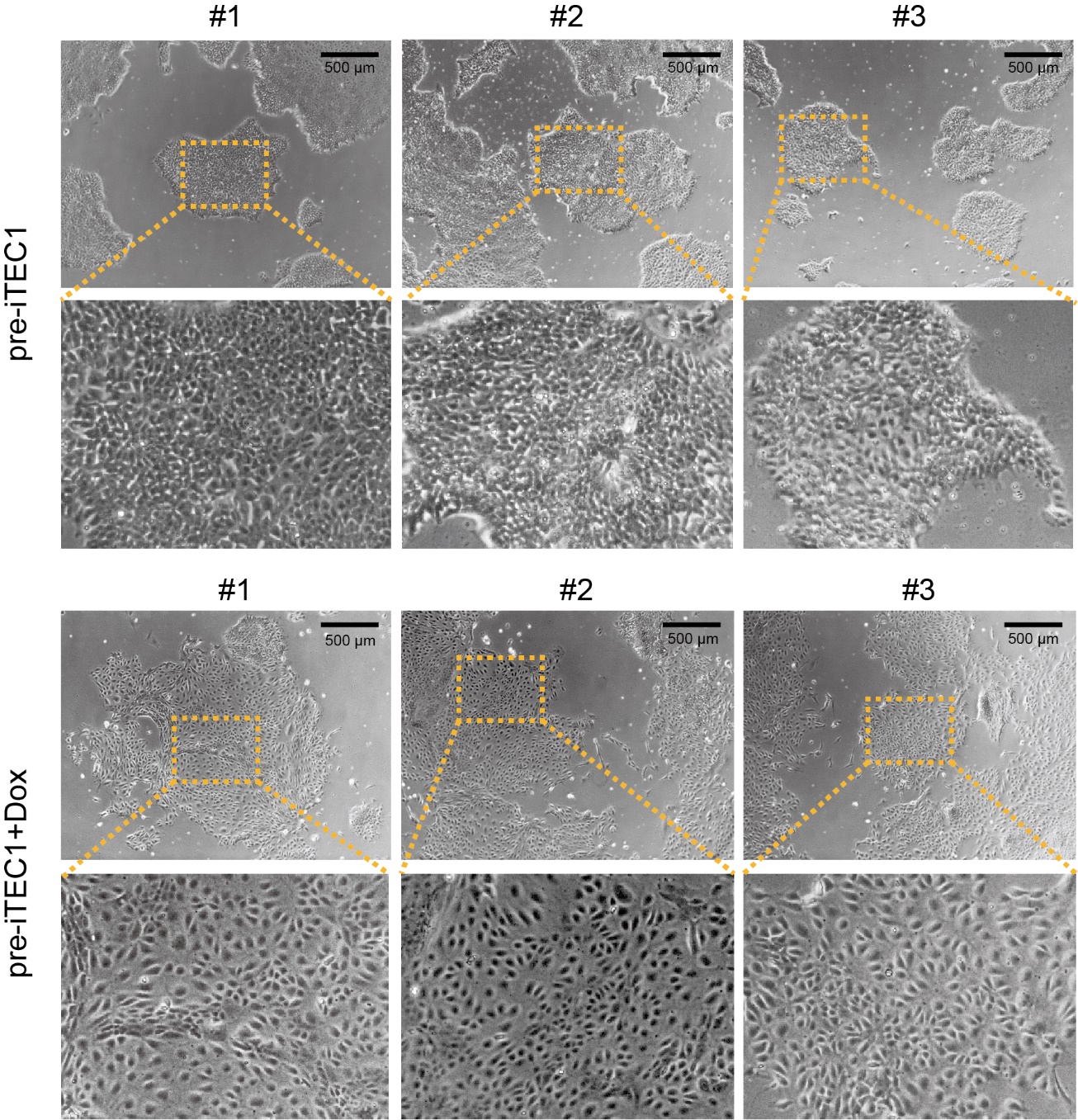


**Supplemental Figure 5. Morphological changes of pre-induced thymic epithelial cells (pre-iTEC) upon doxycycline treatment**

Representative images showing morphological differences between untreated and doxycycline (Dox) -treated pre-iTEC cells. The untreated group (top two rows) maintained a dense colony-like appearance characteristic of iPSCs, whereas the doxycycline-treated group (bottom two rows) exhibited a looser and elongated form.


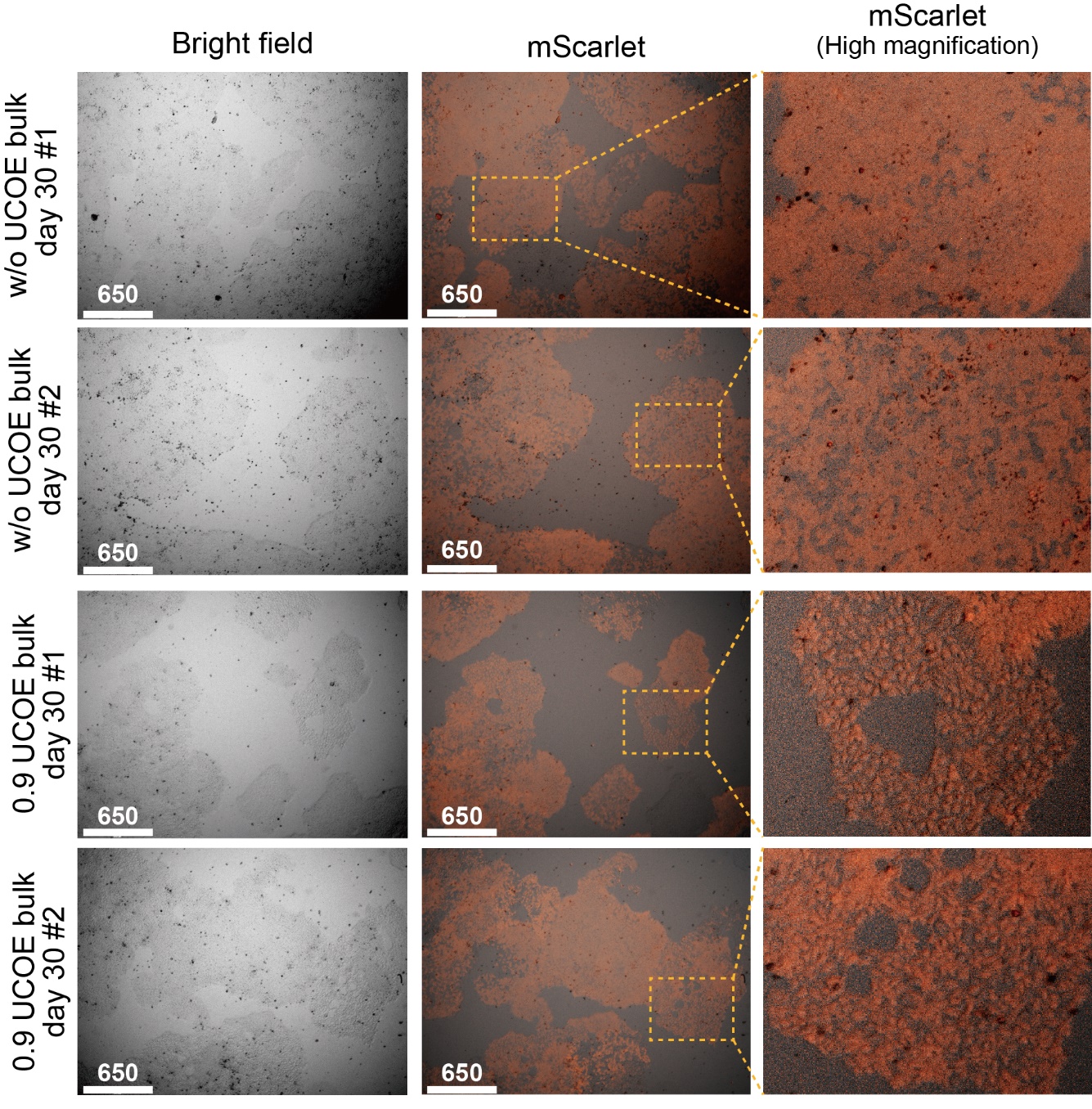


**Supplemental Figure 6. Morphological evaluation of bulk cell populations with integrated 0.9 UCOE-*FOXN1* (0.9 UCOE) or without UCOE-*FOXN1* (w/o UCOE) gene circuits after 30 days of culture.**

Representative images showing bulk cell populations cultured for 30 days in the presence of blasticidin but without doxycycline. The w/o UCOE-*FOXN1* group (top two rows) maintained a dense colony-like appearance, characteristic of iPS cells, even after 30 days. In contrast, the 0.9 UCOE-*FOXN1* group (bottom two rows) exhibited a looser and elongated form.


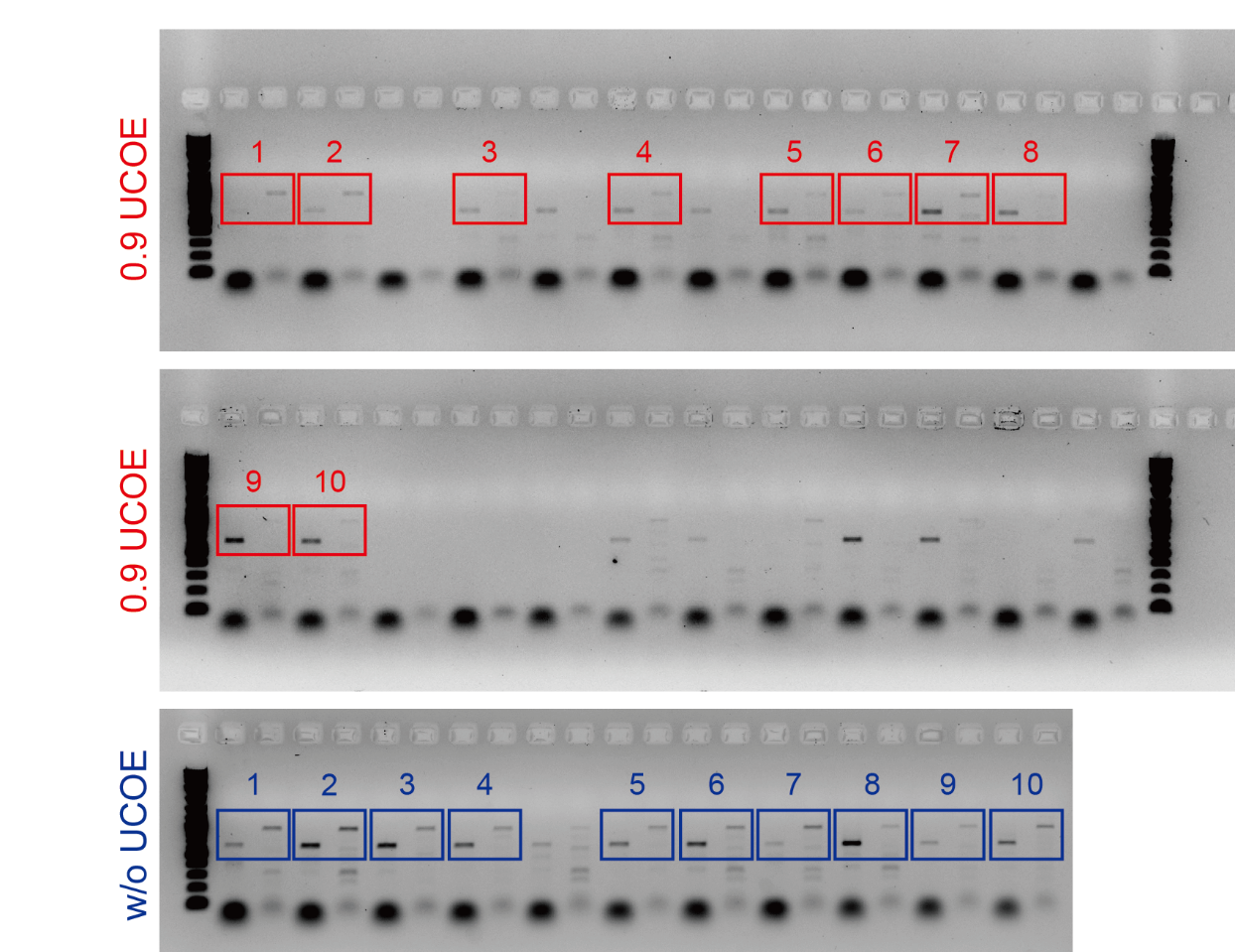


**Supplemental Figure 7. Junction PCR of single-cell-derived clones with integrated 0.9 UCOE-*FOXN1* or without UCOE-*FOXN1* circuits.**

Ten colonies each were picked from the 0.9 UCOE-*FOXN1* (0.9 UCOE) and without UCOE-*FOXN1* (w/o UCOE) groups. PCR was performed to confirm precise gene integration at the Rogi1 target site.


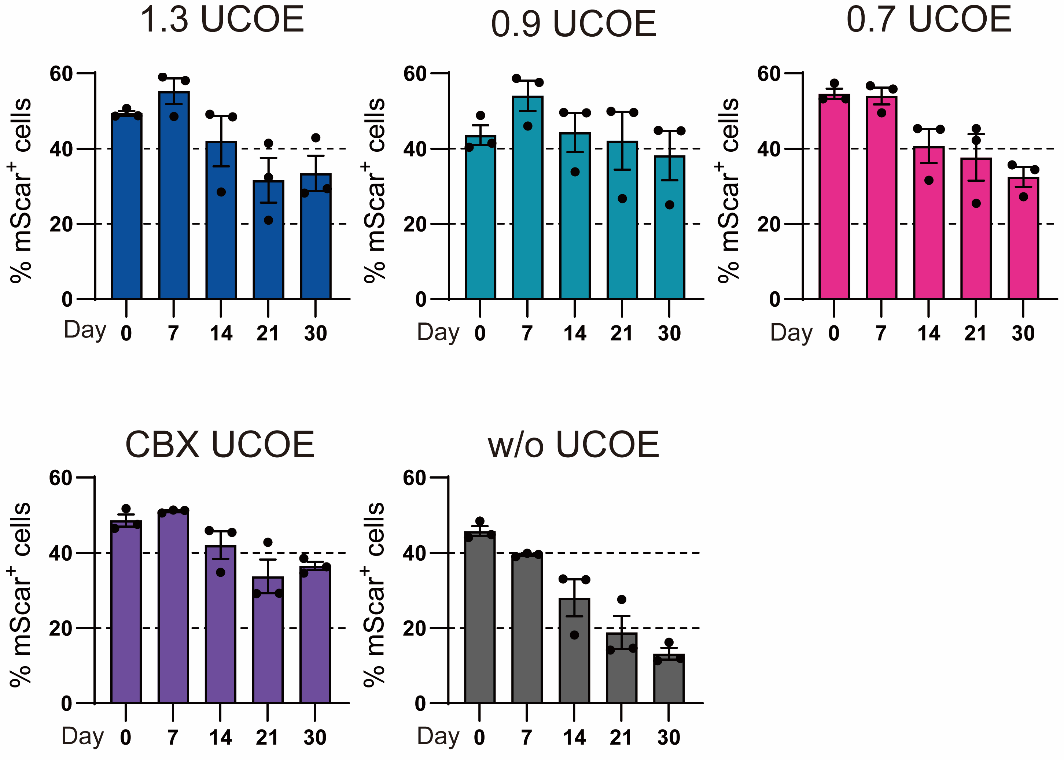


**Supplemental Figure 8. Flow cytometry analysis of RFP-positive cells in various UCOE fragment groups over a 30-day period without blasticidin.**

The RFP-positive (mScarlet+) cell percentage was evaluated in each UCOE fragment group on Days 0, 7, 14, 21, and 30 (n=3).


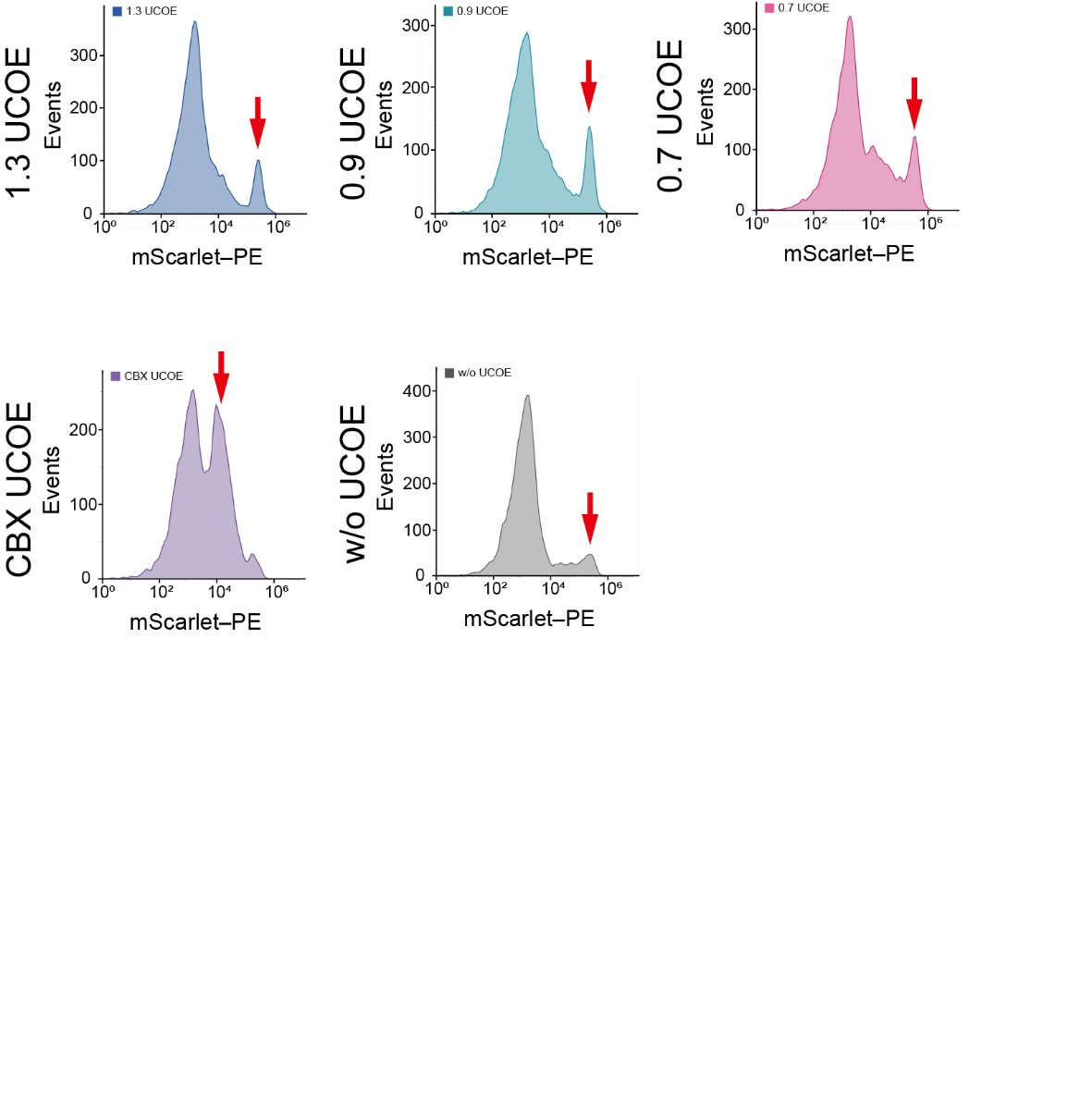

**Supplemental Figure 9. Differences in fluorescence intensity across various UCOE fragments.**

The fluorescence peak for each UCOE fragment group is indicated by a red arrow.


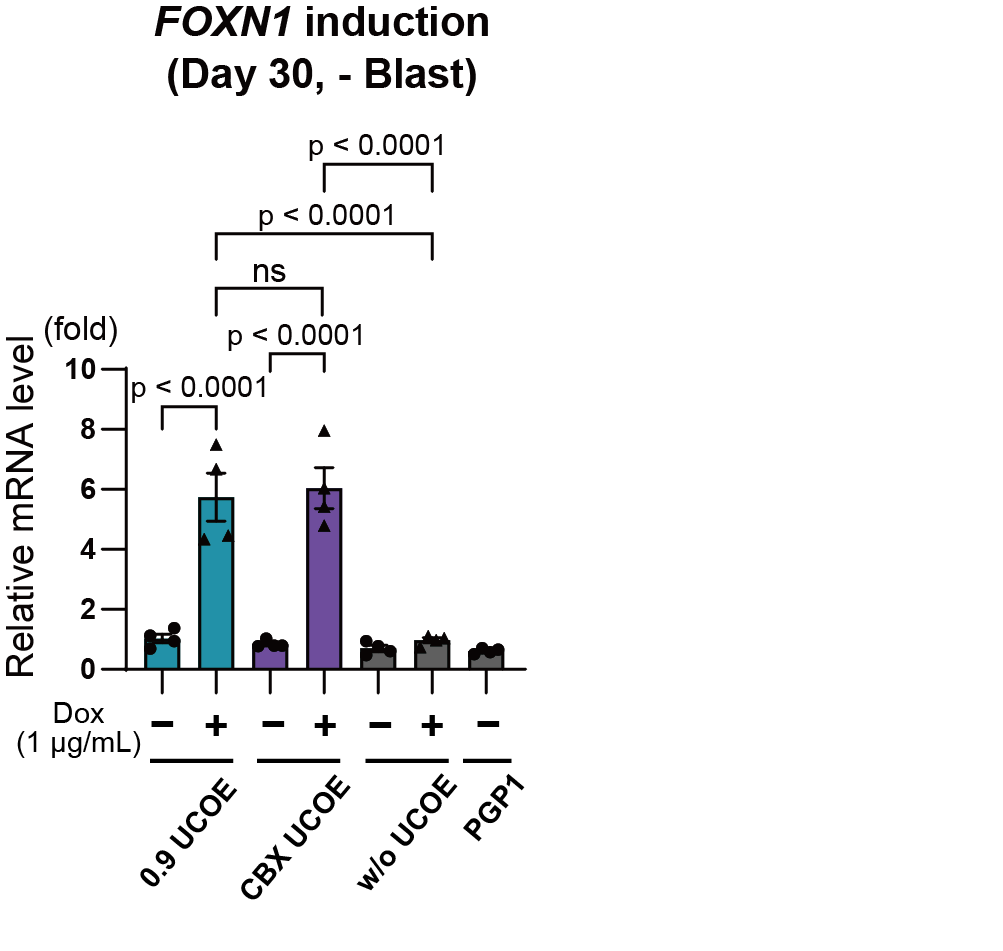


**Supplemental Figure 10. Evaluation of *FOXN1* transcription induction upon doxycycline treatment on Day 30.**

RT-qPCR analysis was used to assess *FOXN1* transcription levels in the 0.9 UCOE, CBX UCOE, and w/o UCOE groups after 30 days of culture in the absence of blasticidin, followed by doxycycline treatment (n=4).

P values were calculated using one-way analysis of variance with Tukey's honestly significant difference test. The data are presented as mean ± SEM. For detailed data, statistical analyses, and exact p-values, see source data file.


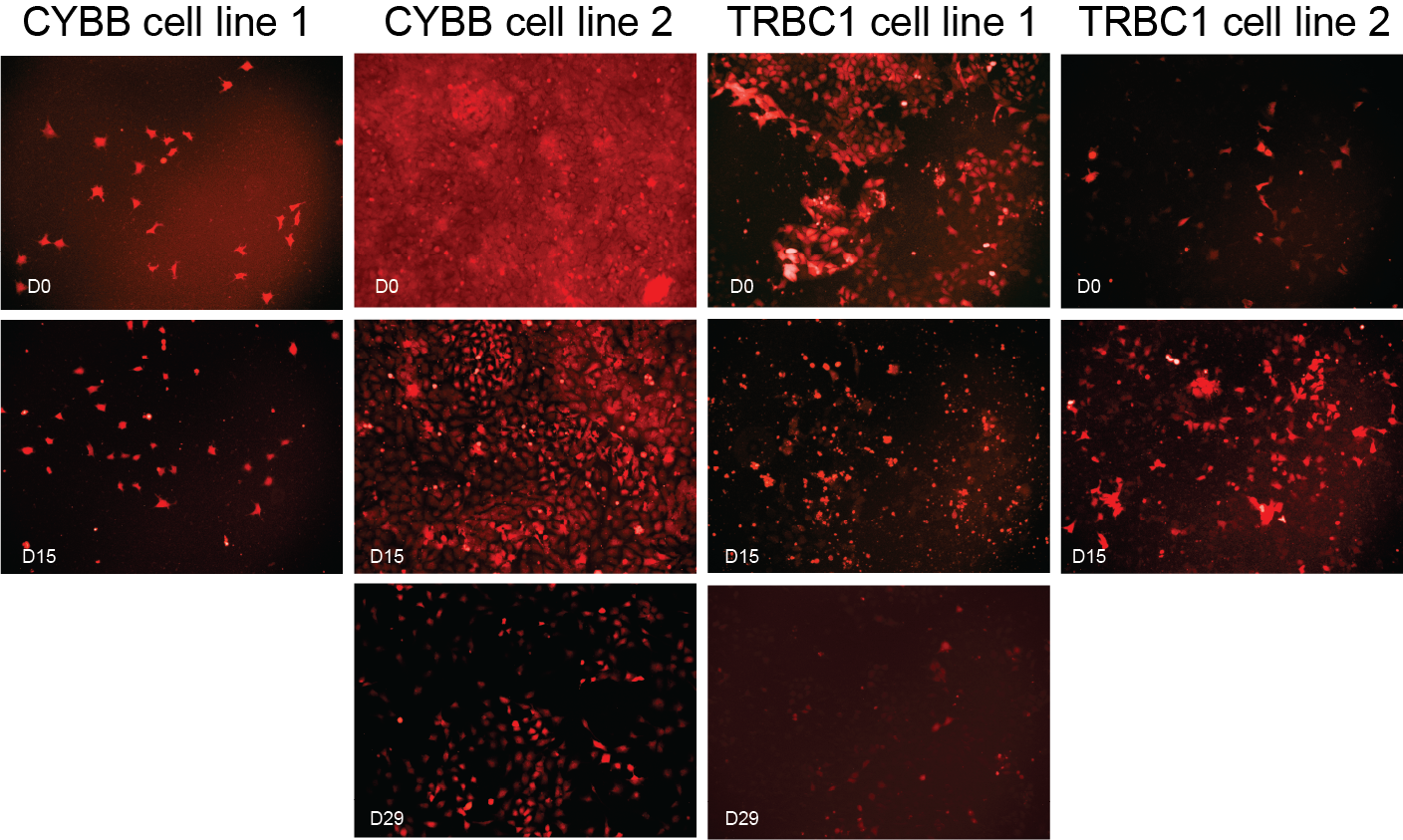


**Supplemental Figure 11. PGP1 iPSC lines with our all-in-one-0.9 UCOE construct integrated into the CYBB and TRBC1 loci.**

Images represent cells cultured in B8 iPSC medium with no blasticidin for 15 - 29 days. Removal of blasticidin leads to differential silencing of RFP (mScarlet) over time, with clone-specific variability in silencing level.


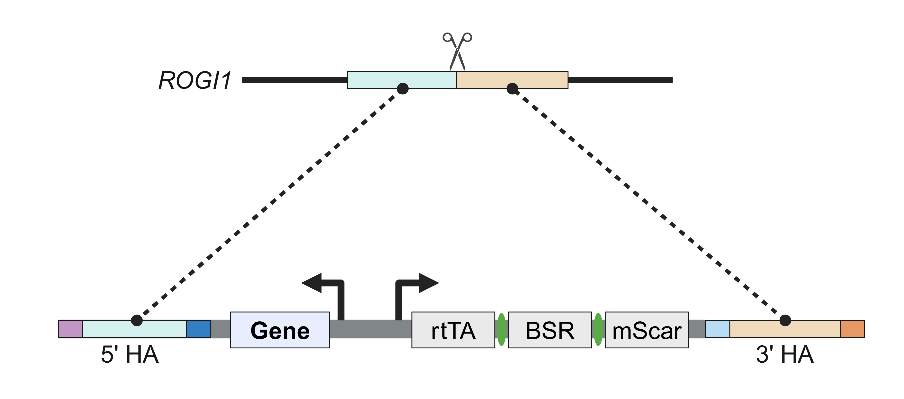


| **Gene name** | **Sequence data (5’ -> 3’)** |
| --- | --- |
| CRISPR site of Rogi1 region | GTGCAAGCATTGATGCAACTTACTGATAAAGGTGAAACTGTAAAGTATACTTTAAAATAGATGCAGTAAGTAGAATTAGAGTTAGCTTCCATCACCTTTTAATCTACAAATGATTTTACAGAGAAAGCAGCATTAAAGATCTTTGTGGGCAATCAAAACAGTAATTTGAGAATAGCATTATACACTGCATTTAAGTAGGATTCAATAATTTTAAAGTGCAGGGACAAAATTTCCTCATATGGCTCACTAGCTACATTGCAAATTTCTTGAAATCAGAACACAGAAGTGCAGTCCTGTGCTCGCAATGCAGACTTGCAGGGTGTAGAGGCATAAATGGCTCCAGAGCCAGGGACATGGGTCCAGAGGGGGGTAGTCTCCAGAAGACTCCTTTCGGGCCTATTACCATGCCTCAGAGGTCCAAGTGGGGCATGGTGAATATATTATCCTTTATATTATATTTCTTATATGTCTACAACTGCCACTT*CATGGCACTAGGACTAAAGGTTGGCCAAAGTACAAGATATTTGTCTTATCTGATGACAACTCTGTGTCCTGGACTCTCTTCCAGAATAAGACCTTTCCTGCAGCACTGCTTGAACTCCTCTTAGCAAGAGGGAAACATGTGAAATGCTACCAAAATAGAATAGAAGTAAATTCTTATTATATTCCTTTGTTCACTCATATCCTGAAGTGCATCAAATCAGGTTTTCTCACCTGTATAATGCTGTATTTTACTTGAGTTGGAATAATTTTGCTTAGAAATAAATAAGTAAAACAGCACCTGCCTCCAGACCTAGGGTCCATCAGGAAAAATATAAGGTATATGAGGTGTATGCTCTAAACCCAAGGCCAACATGTATAGGAAAACCTTAAGTCCTTCAGTGCATGTGCTTGGATGAAGAAGGTAATACAATTGTAGGCAACTGCAAGAGCAATGTAGGTAAAATTCACACCTACAGGCAGTCGTGAAAAT |
| 5’ HA | GTGCAAGCATTGATGCAACTTACTGATAAAGGTGAAACTGTAAAGTATACTTTAAAATAGATGCAGTAAGTAGAATTAGAGTTAGCTTCCATCACCTTTTAATCTACAAATGATTTTACAGAGAAAGCAGCATTAAAGATCTTTGTGGGCAATCAAAACAGTAATTTGAGAATAGCATTATACACTGCATTTAAGTAGGATTCAATAATTTTAAAGTGCAGGGACAAAATTTCCTCATATGGCTCACTAGCTACATTGCAAATTTCTTGAAATCAGAACACAGAAGTGCAGTCCTGTGCTCGCAATGCAGACTTGCAGGGTGTAGAGGCATAAATGGCTCCAGAGCCAGGGACATGGGTCCAGAGGGGGGTAGTCTCCAGAAGACTCCTTTCGGGCCTATTACCATGCCTCAGAGGTCCAAGTGGGGCATGGTGAATATATTATCCTTTATATTATATTTCTTATATGTCTACAACTGCCACTT |
| 3’ HA | CATGGCACTAGGACTAAAGGTTGGCCAAAGTACAAGATATTTGTCTTATCTGATGACAACTCTGTGTCCTGGACTCTCTTCCAGAATAAGACCTTTCCTGCAGCACTGCTTGAACTCCTCTTAGCAAGAGGGAAACATGTGAAATGCTACCAAAATAGAATAGAAGTAAATTCTTATTATATTCCTTTGTTCACTCATATCCTGAAGTGCATCAAATCAGGTTTTCTCACCTGTATAATGCTGTATTTTACTTGAGTTGGAATAATTTTGCTTAGAAATAAATAAGTAAAACAGCACCTGCCTCCAGACCTAGGGTCCATCAGGAAAAATATAAGGTATATGAGGTGTATGCTCTAAACCCAAGGCCAACATGTATAGGAAAACCTTAAGTCCTTCAGTGCATGTGCTTGGATGAAGAAGGTAATACAATTGTAGGCAACTGCAAGAGCAATGTAGGTAAAATTCACACCTACAGGCAGTCGTGAAAAT |
| gRNA | TTAGTCCTAGTGCCATGAAG |

**Supplemental Table 1. Sequence information for homology arms and gRNA for genomic integration at the Rogi1 site.**
This table provides the sequence information for the homology arms and gRNA used for CRISPR/Cas9-mediated cleavage at the Rogi1 site. The cleavage site is marked with an asterisk (*). The 5' homology arm region is indicated by a green underline, and the 3' homology arm region by a yellow underline.


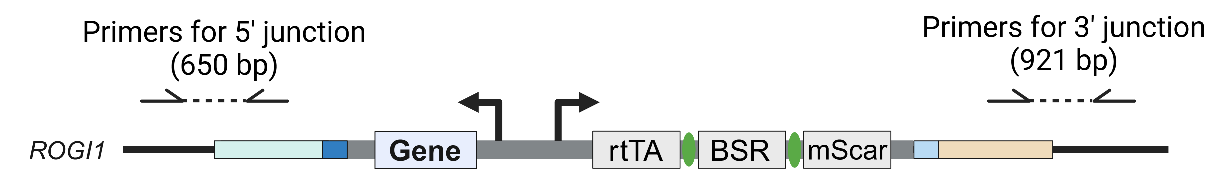


| **Primer name** | **Sequence data (5’ -> 3’)** |
| --- | --- |
| Forward primer for 5’ junction | ACACACATGACTCAGTCAGAACTGCA |
| Reverse primer for 5’ junction | ATGTCTGGGTCGACGAAGTT |
| Forward primer for 3’ junction | CCACTCCCACTGTCCTTTCC |
| Reverse primer for 3’ junction | TGGCACTACTTTTTCGCAGC |

**Supplemental Table 2. Primer sequences for junction PCR following genomic integration.**
This table lists the primer sequences used for junction PCR at the 5' and 3' junctions following genomic integration.

| **Gene** | **Fwd (5’>3’)** | **Rev (5’>3’)** |
| --- | --- | --- |
| *FOXN1* | CAAAACATGCCAGCGCCGCTTT | GCCTGGTGCAATGGACTGCCTT |
| *DLL4* | CGGGTACCTTCTCGCTCATC | ATGAGTGCATCTGGTGGCAA |
| Ly51 | ATGGGAAGAACATGGCCTGG | AAGCTCTCCATCTGCCACAG |
| *RPS29* | AATATGTGCCGCCAGTGTTT | CCCGGATAATCCTCTGAAGG |
| rtTA | TACACTGGGCTGCGTATTGG | CCGCTTTCGCACTTTAGCTG |
| BSR | GTGCAGAAGCCATTGCGATT | ACCACAAGGACTTACCACTCG |
| mScarlet | CACGAGTTCGAGATCGAGGG | GCCGTCCTCGAAGTTCATCA |
| *OCT4* | AGTGAGAGGCAACCTGGAGA | ACACTCGGACCACATCCTTC |
| *NANOG* | GAGATGCCTCACACGGAGAC | GGGTTGTTTGCCTTTGGGAC |
| *DNMT3B* | GACTCGATCCTCGTCAACGG | ATCTTCGGCCTCTGATCTCCG |

**Supplemental Table 3. Primer sequences used for qPCR analysis.**
This table provides the sequences of primers used in qPCR experiments.

| 5’ -> 3’ |
| --- |
| **0.9 UCOE (863 bp)**:  TGAAATTAACGCCGACGGGAGGGGCTTAATCCGCAGCCTGGAGATCCAGCCCCCTCAACCCGGGAGGTGGTCCCTGCAGTTACGCCAATGATAACCCCCGCCAGAAAAATCTTAGTAGCCTTCCCTTTTTGTTTTCCGTGCCCCAACTCGGCGGATTGACTCGGCCCCTTCCGGAAACACCCGAATCAACTTCTAGTCAAATTATTGTTCACGCCGCAATGACCCACCCCTGGCCCGCGTCTGTGGAACTGACCCCTGGTGTACAGGAGAGTTCGCTGCTGAAAGTGGTCCCAAAGGGGTACTAGTTTTTAAGCTCCCAACTCCCCCTCCCCCAGCGTCTGGAGGATTCCACACCCTCGCACCGCAGGGGCGAGGAAGTGGGCGGAGTCCGGTTTTGGCGCCAGCCGCTGAGGCTGCCAAGCAGAAAAGCCACCGCTGAGGAGACTCCGGTCACTGTCCTCGCCCCGCCTCCCCCTTCCCTCCCCTTGGGGACCACCGGGCGCCACGCCGCGAACGGTAAGTGCCGCGGTCGTCGGCGCCTCCGCCCTCCCCCTAGGGCCCCAATTCCCAGCGGGCGCGGCGCGCGGCCCCTCCCCCCGCCGGGCGCGCGCCCGCTGCCCCGCCCTTCGTGGCCGCCCGGCGTGGGCGGTGCCACCCCTCCCCCCGGCGGCCCCGCGCGCAGCTCCCGGCTCCCTCCCCCTTCGGATGTGGCTTGAGCTGTAGGCGCGGAGGGCCGGAGACGCTGCAGACCCGCGACCCGGAGCAGCTCGGAGGCGGTGAAGTCGGTGGCTTTCCTTCTCTCTAGCTCTCGCTCGCTGGTGGTGCTTCAGATGCCACACGCGTCCCGGGGGCCCGGTTCTCCG |
| **1.3 UCOE (1337 bp):**  TCCATCGCGGACTCAGTCGCTTCAGCCCGATTTCCCGCAGCCGAGCGAGATGAGAGAGATCTCCGCGGACGAACACGAACCGGACTCGTCCTGGCGCTGTAGTGAGAACTGCCGCTGCTCGAGAAACAACTCTGCGAGGAGCACCTCCGCACGGGACCCGGCGCTGCTGCTACTGCCGCTAGAGCCGCTGCCGCCGCTTTTCTAGAACCTTCCCCCCCACTAACGCGTCTTCCGCTACGTCAGGCCGTCGCGTAAACGCCCTATCCGCCGCCAATGGCGGGAAGGCTCTACGCCCCACCTTACGCCAAATGCGTACTCCTCCCACCCTTGCGGCCAGAGACAGTACCCGACGTTACTTCCGTAAATGCGCTCAATGAATTGCGGAAGGCTAGAGTCCTGCTAGTTACTACCTCTTGGAATAGGGTCCCGCCCCCTGCCTTGGCGCAAGGCAGGTGAGAAACGGTCGCGCAGTTTGAAATTAACGCCGACGGGAGGGGCTTAATCCGCAGCCTGGAGATCCAGCCCCCTCAACCCGGGAGGTGGTCCCTGCAGTTACGCCAATGATAACCCCCGCCAGAAAAATCTTAGTAGCCTTCCCTTTTTGTTTTCCGTGCCCCAACTCGGCGGATTGACTCGGCCCCTTCCGGAAACACCCGAATCAACTTCTAGTCAAATTATTGTTCACGCCGCAATGACCCACCCCTGGCCCGCGTCTGTGGAACTGACCCCTGGTGTACAGGAGAGTTCGCTGCTGAAAGTGGTCCCAAAGGGGTACTAGTTTTTAAGCTCCCAACTCCCCCTCCCCCAGCGTCTGGAGGATTCCACACCCTCGCACCGCAGGGGCGAGGAAGTGGGCGGAGTCCGGTTTTGGCGCCAGCCGCTGAGGCTGCCAAGCAGAAAAGCCACCGCTGAGGAGACTCCGGTCACTGTCCTCGCCCCGCCTCCCCCTTCCCTCCCCTTGGGGACCACCGGGCGCCACGCCGCGAACGGTAAGTGCCGCGGTCGTCGGCGCCTCCGCCCTCCCCCTAGGGCCCCAATTCCCAGCGGGCGCGGCGCGCGGCCCCTCCCCCCGCCGGGCGCGCGCCCGCTGCCCCGCCCTTCGTGGCCGCCCGGCGTGGGCGGTGCCACCCCTCCCCCCGGCGGCCCCGCGCGCAGCTCCCGGCTCCCTCCCCCTTCGGATGTGGCTTGAGCTGTAGGCGCGGAGGGCCGGAGACGCTGCAGACCCGCGACCCGGAGCAGCTCGGAGGCGGTGAAGTCGGTGGCTTTCCTTCTCTCTAGCTCTCGCTCGCTGGTGGTGCTTCAGATGCCACACGCGTCCCGGGGGCCCGGTTCTCCG |
| **0.7 UCOE (749 bp):**  GTAGCCTTCCCTTTTTGTTTTCCGTGCCCCAACTCGGCGGATTGACTCGGCCCCTTCCGGAAACACCCGAATCAACTTCTAGTCAAATTATTGTTCACGCCGCAATGACCCACCCCTGGCCCGCGTCTGTGGAACTGACCCCTGGTGTACAGGAGAGTTCGCTGCTGAAAGTGGTCCCAAAGGGGTACTAGTTTTTAAGCTCCCAACTCCCCCTCCCCCAGCGTCTGGAGGATTCCACACCCTCGCACCGCAGGGGCGAGGAAGTGGGCGGAGTCCGGTTTTGGCGCCAGCCGCTGAGGCTGCCAAGCAGAAAAGCCACCGCTGAGGAGACTCCGGTCACTGTCCTCGCCCCGCCTCCCCCTTCCCTCCCCTTGGGGACCACCGGGCGCCACGCCGCGAACGGTAAGTGCCGCGGTCGTCGGCGCCTCCGCCCTCCCCCTAGGGCCCCAATTCCCAGCGGGCGCGGCGCGCGGCCCCTCCCCCCGCCGGGCGCGCGCCCGCTGCCCCGCCCTTCGTGGCCGCCCGGCGTGGGCGGTGCCACCCCTCCCCCCGGCGGCCCCGCGCGCAGCTCCCGGCTCCCTCCCCCTTCGGATGTGGCTTGAGCTGTAGGCGCGGAGGGCCGGAGACGCTGCAGACCCGCGACCCGGAGCAGCTCGGAGGCGGTGAAGTCGGTGGCTTTCCTTCTCTCTAGCTCTCGCTCGCTGGTGGTGCTTCAGATGCCACACGCGTCCCGGGGGCCCGGTTCTCCG |
| **CBX UCOE (547 bp):**  CCAACTCCCCCTCCCCCAGCGTCTGGAGGATTCCACACCCTCGCACCGCAGGGGCGAGGAAGTGGGCGGAGTCCGGTTTTGGCGCCAGCCGCTGAGGCTGCCAAGCAGAAAAGCCACCGCTGAGGAGACTCCGGTCACTGTCCTCGCCCCGCCTCCCCCTTCCCTCCCCTTGGGGACCACCGGGCGCCACGCCGCGAACGGTAAGTGCCGCGGTCGTCGGCGCCTCCGCCCTCCCCCTAGGGCCCCAATTCCCAGCGGGCGCGGCGCGCGGCCCCTCCCCCCGCCGGGCGCGCGCCCGCTGCCCCGCCCTTCGTGGCCGCCCGGCGTGGGCGGTGCCACCCCTCCCCCCGGCGGCCCCGCGCGCAGCTCCCGGCTCCCTCCCCCTTCGGATGTGGCTTGAGCTGTAGGCGCGGAGGGCCGGAGACGCTGCAGACCCGCGACCCGGAGCAGCTCGGAGGCGGTGAAGTCGGTGGCTTTCCTTCTCTCTAGCTCTCGCTCGCTGGTGGTGCTTCAGATGCCACACGCGTCCCGGGGGCCCGGTTCTCCG |

**Supplemental Table 4. Sequence information for various A2UCOE fragments**

This table provides the sequence information for different A2UCOE fragments, including 0.9 UCOE, 1.3 UCOE, 0.7 UCOE, and CBX UCOE.

|  | 5’ -> 3’ |
| --- | --- |
| AT35 (36.9%) | TCGCCGCGATGGTGGGCGCTTTGTATTTGCGTGCCCGCGTTGGGCCGTCGAGTCGGCATTTGAGCCACCGGGCCTTGAGTGGCCCAGCTCTGCCCATCACAAGACCACCGGATCCAGCAGGCGTATGTGGAAGGCGTGGGTTCACTGCGGGTAATGGCTAAGTACGCGCGCGCCTATGCGGCGCGAATCGGAGCATATGTCCAAGCCGGTCGGTCCGACGAAGCGACCGTGTGAACTT |
| AT50  (52.5%) | ACCGATAGATACTGTCGCTAGGTCGTAGCTAAATTGATCGGGTCCACCGATAGTTACATTTACGCCACAGCGACGCTTGGCCCTAGTTCACTAGCGCTCCGTCTCAACGTTTGAGTCCTTTGTGACCTCACATCATTGGCTATTGCGGTTCTTAAAAACAATGTCATCTTGACGACTGAAGTTATGCGGTACAGCGAAAGAACAGTGATAGCGTAACTCAGTATACGAATCCCGCGAC |
| AT65  (63.0%) | CGATTATTAGCGTTATCTATTCGATTCCGTGTAATTTCGTTTTTTGGTCATTGAAATCAACAAGATCCGGGCCGAACTTCAGATAGTTAGATACTCCTCTCAGCACCGTTTGGCTTGATTTAATCTCACACGATGAGATCTCCGACATTCAGGTCAAATCTTAACCAATGGAGTAGAAGGCAAACTCACTTTTTATCAAAGTTTATCATTTATTGAATTGTAAAGTGACAATTCCTCA |
| SV40  (66.1%) | TAATCAGCCATACCACATTTGTAGAGGTTTTACTTGCTTTAAAAAACCTCCCACACCTCCCCCTGAACCTGAAACATAAAATGAATGCAATTGTTGTTGTTAACTTGTTTATTGCAGCTTATAATGGTTACAAATAAAGCAATAGCATCACAAATTTCACAAATAAAGCATTTTTTTCACTGCATTCTAGTTGTGGTTTGTCCAAACTCATCAATGTATCTTATCATGTCTGG *SV40 polyA region |

**Supplemental Table 5. Sequence information for random AT-rich spacer sequences.**

This table provides the sequence information for the AT35, AT50, AT65, and SV40 spacer sequences. The SV40 polyadenylation (poly A) region is highlighted in red.
